# Multiple Perspectives on the Need for Real‐World Evidence to Inform Regulatory and Health Technology Assessment Decision‐Making: Scoping Review and Stakeholder Interviews

**DOI:** 10.1002/pds.70074

**Published:** 2025-01-07

**Authors:** Marieke S. Jansen, Olaf M. Dekkers, Saskia le Cessie, Lotty Hooft, Helga Gardarsdottir, Anthonius de Boer, Rolf H. H. Groenwold

**Affiliations:** ^1^ Department of Clinical Epidemiology Leiden University Medical Center Leiden The Netherlands; ^2^ Department of Endocrinology and Metabolic Disorders Leiden University Medical Center Leiden The Netherlands; ^3^ Dutch Medicines Evaluation Board Utrecht The Netherlands; ^4^ Department of Biomedical Data Sciences Leiden University Medical Center Leiden The Netherlands; ^5^ Cochrane Netherlands, University Medical Center Utrecht Utrecht The Netherlands; ^6^ Julius Center for Health Sciences and Primary Care, University Medical Center Utrecht Utrecht The Netherlands; ^7^ Division of Pharmacoepidemiology and Clinical Pharmacology Utrecht Institute for Pharmaceutical Sciences, University Utrecht Utrecht The Netherlands; ^8^ Department of Clinical Pharmacy University Medical Center Utrecht Utrecht The Netherlands; ^9^ Faculty of Pharmaceutical Sciences University of Iceland Reykjavik Iceland

**Keywords:** health technology assessment, real‐world data, real‐world evidence, regulatory science

## Abstract

**Purpose:**

Real‐world evidence (RWE) is increasingly considered in regulatory and health technology assessment (HTA) decision‐making, though perspectives on its relevance may vary. Expanding on a recent review regarding regulatory decisions, this study aimed to identify factors influencing the need for RWE in HTA decision‐making, confirm and enrich factors with stakeholder views, and evaluate similarities and differences between regulatory and HTA needs.

**Methods:**

Previous scoping review methodology was used to identify factors influencing the need for RWE in HTA decision‐making. Semi‐structured interviews with stakeholders were conducted to confirm and enrich literature‐derived factors for both regulatory and HTA contexts. Insights from the reviews and interviews were combined to explore similarities and differences in RWE needs across these domains.

**Results:**

The HTA review, featuring 118 articles, revealed two major themes and six subthemes, encompassing 45 factors. The need for RWE depended on (1) questions addressable with RWE, and (2) contextual factors. Stakeholder interviews confirmed literature‐derived factors. While contextual factors aligned between regulatory and HTA decision‐making, question‐related factors partly differed. Unlike the benefit–risk assessment in regulatory decision‐making, RWE serves as direct input for the HTA, and involves specific details and a broader scope. Regulators require RWE for orphan status submissions, alternative approval pathways and to evaluate the impact of risk minimization measures, whereas HTA uses RWE to guide comparator selection, evaluate treatment implementation, quality of care and general healthcare impacts.

**Conclusion:**

Contextual factors that influence the need for RWE are similar between regulatory and HTA decision‐making, with variations seen in questions addressable with RWE.

**Summary**

- Although increasingly considered in decision‐making, the relevance of real‐world evidence (RWE) differs between regulatory and health technology assessment (HTA).
- This study analysed factors influencing the need for RWE to inform decision‐making, comparing them across the regulatory and HTA domain.
- The need for RWE was found to depend on the questions addressable with RWE, and various contextual factors. While contextual factors aligned between the regulatory and HTA domain, the questions differed, predominantly due to the broader scope of HTA.
- This overview may help stakeholders recognize opportunities where RWE can serve evidentiary needs of both regulatory and HTA decision‐makers.

## 1 Introduction

Real‐world evidence (RWE) is increasingly recognized as a valuable addition to results of traditional trials, both by regulators to inform market authorization decisions, and by health technology assessment (HTA) decision‐makers to inform reimbursement recommendations. While regulators focus primarily on product's efficacy and safety (i.e., benefit–risk assessment) for market approval, HTA decision‐makers consider a broader scope, including cost‐effectiveness, performance relative to existing treatments, and societal value, to determine whether and how a product or technology should be funded and used within a healthcare system. RWE may not only help inform these decisions, but could also serve as a valuable tool to evaluate their subsequent impact. Over the last decade, regulatory agencies and HTA bodies, among others, have developed various RWE frameworks and guidance documents that aim to improve the quality of RWE [1, 2, 3, 4, 5, 6]. However, it remains unclear in which scenarios RWE could best be leveraged to aid decision‐making, particularly within the regulatory context [7, 8].

To address this knowledge gap, we recently conducted a scoping review to identify factors reported in literature that make RWE necessary or desirable to inform regulatory decision‐making [9]. The need for RWE was found to depend on two overarching themes, being the nature of questions that need to be answered in order to facilitate regulatory decision‐making, and contextual factors related to the feasibility and ethical considerations regarding traditional randomized trials. Additionally, limitations of available evidence, as well as disease and treatment specific aspects contribute to the need for RWE [9].

While these findings provide insights into the circumstances where RWE can aid regulatory decision‐making, these circumstances could be different for HTA decision‐making. For example, the broader scope of HTA, extending beyond benefit–risk and including dimensions such as cost‐effectiveness and relative effectiveness, likely results in an inherently greater need for RWE. Identifying differences and similarities in factors influencing the need for RWE in regulatory and HTA contexts could aid in optimizing evidence generation processes during drug development. What is more, further validation of the factors and themes by stakeholders would be valuable as RWE discussions evolve rapidly, and the views described in literature might be incomplete.

In the present study, our goal was to complement and expand on the findings of the previous scoping review addressing the need for RWE in regulatory decision‐making [9]. Specifically, we aimed to: (1) Identify factors reported in literature that influence the need or desire for RWE in HTA decision‐making, (2) confirm and enrich factors from literature with stakeholder views for both regulatory and HTA decision‐making, and (3) evaluate how these factors, as well as the eventual need for RWE, overlap and differ between regulatory and HTA decision‐making.

## 2 Methods

Previously, we conducted a comprehensive scoping review on the need for RWE in regulatory decision‐making [9]. In this study we extend that work, by (1) conducting a scoping review on HTA decision‐making; (2) conducting semi‐structured interviews on regulatory and HTA decision‐making with various stakeholders; and (3) comparing the need for RWE between the regulatory and HTA domain. Here, RWE is defined as information derived from the analysis of real‐world data (RWD), which refers to data relating to a patient's health status or the delivery of healthcare collected routinely from a variety of sources other than traditional clinical trials [2]. We used information from the previous regulatory‐focused review, to inform methodological components of the HTA scoping review and stakeholder interviews in the present study [9]. The findings from both scoping reviews and the stakeholder interviews were used to draw comparisons between regulatory and HTA decision‐making. Details on sources of information and methods are described below. We used the PRISMA‐ScR statements and Consolidated Criteria for Reporting Qualitative Research (COREQ) for reporting our research [10, 11].

### 2.1 HTA Scoping Review

The methodology of the previous regulatory‐focused review, including the selection of articles, was used to now identify factors regarding the need for RWE in HTA decision‐making. A comprehensive overview of the scoping review methodology has been described previously [9]. In short, we conducted a search in five electronic databases (PubMed, Embase, Emcare, Web of Science, Cochrane Library) for articles addressing RWE in regulatory and HTA decision‐making. This search was conducted in November 2022 and did not include restrictions for a specific time period. Additionally, we searched official websites of regulatory agencies, HTA bodies, and research institutes or other professional organizations involved with RWE, for relevant information (e.g., white papers, frameworks, guidance documents, guidelines—henceforth these are also referred to as articles). This grey literature search was conducted in February 2023. Articles were included if they: (1) Discussed factors or contexts that impact whether RWE could be necessary or desirable in regulatory or HTA decision‐making; (2) focused on pharmacological or biological interventions in humans; and (3) considered perspectives on Europe or North‐America, or without a focus on a specific region. Conference abstracts and presentations were not considered. Articles published in languages other than English or Dutch were also excluded.

Eligible articles were included in the review and read full‐text. A thematic synthesis approach was then used to analyse the content of all included articles, in order to identify contextual factors influencing the necessity or desirability of RWE in HTA decision‐making [12].

### 2.2 Stakeholder Interviews

#### 2.2.1 Study Design and Population

We also conducted semi‐structured interviews with representatives of stakeholders involved with regulatory and HTA decision‐making. To obtain a wide range of perspectives on both regulatory and HTA decision‐making, we approached regulators, health technology assessors, academia, pharmaceutical industry, data providers, technology providers, clinicians and patient advocates, located in Europe or North‐America. Participants had to have some form of experience with regulatory or HTA decision‐making, but no other eligibility criteria were applied. Participants were identified through a convenience sample, and consisted of the research team's network and people suggested by members of the research team's network. Standardized emails were sent to invite potential candidates, as well as a reminder after two weeks in case no response was received; no further reminders were sent.

#### 2.2.2 Interview Guide Development

The interview guide was developed based on the preliminary results of both scoping reviews (i.e., the previously published regulatory review, and the HTA review presented in this paper), and aimed to confirm and enrich literature‐derived factors that increase the need for RWE in regulatory and HTA decision‐making [9]. Thus, both the regulatory and the HTA scope were discussed during the interviews. The key question of the interview was “When do you think RWE could be necessary, or desirable, to inform regulatory or HTA decision‐making?” To help elicit views and ideas regarding this question, a scheme of a medicine's lifecycle was shown which included various phases (e.g., a pre‐approval phase with (pre‐) clinical development, regulatory and HTA review, and a post‐approval phase with potential post‐approval obligations, monitoring, and label expansions) (Figure S1). Here, the potentially varying need for RWE across a medicine's lifecycle was also discussed. Finally, participants were asked if they recognized some of the themes identified in the scoping reviews, such as generalizability, feasibility and ethical considerations, if not already discussed spontaneously during the earlier parts of the interview (see Table S1 for an overview of these themes). Participants were explicitly asked to share their personal views (and thus not on behalf of an institution or organization). Two pilot interviews were held to refine the content of the interview guide.

#### 2.2.3 Data Collection

In depth semi‐structured interviews were conducted in April–May 2023, by a single researcher (MJ), or two researchers (MJ and RG) at a time. Two participants were interviewed simultaneously, while the others were interviewed alone. Interviews lasted between 45 and 90 min, and were conducted online or via phone call, based on participant preference. After a brief introduction of the project, verbal consent was obtained before the start of the interview. All interviews were audio‐recorded and subsequently transcribed using Whisper and manual corrections where necessary [13]. Transcripts were pseudonymized before analysis.

#### 2.2.4 Data Analysis

We used a thematic synthesis approach to analyse the transcripts [12]. As changes to the interview guide were made only after the first pilot interview, and not after the second, we also included the transcript from the second pilot interview in the analysis. Data were summarized with line‐by‐line coding, using descriptive and interpretative approaches [12]. Codes were then refined through an iterative process of revisiting transcripts, and partly categorized under predefined themes identified in the scoping reviews (deductive coding). The predefined themes included all themes and factors identified in the regulatory and HTA scoping review. Codes that did not fit into this existing framework, were categorized under newly emerging themes derived from the data (inductive coding).

Initial coding and analysis was performed by a single researcher (MJ), using ATLAS.ti software (GmbH, Berlin, version 23.2.2.27458). Resulting themes and subthemes were then discussed within the research team to assess consistency of interpretation, and were refined if needed.

Following the initial analysis of the stakeholder interviews, factors identified from the interviews were compared with those identified from the literature, which encompassed both scoping reviews: (i) Regulatory domain (reported previously), and (ii) HTA domain (presented in this paper). This comparison aimed to evaluate whether stakeholder views corresponded with, and potentially enriched, factors and themes identified from the literature.

### 2.3 Comparison Between Regulatory and HTA Decision‐Making

The final aim of the current study was to evaluate how factors that influence the need or desire for RWE may differ between regulatory and HTA decision‐making. To facilitate this comparison, the collective findings from both scoping reviews and the stakeholder interviews were summarized into two distinct frameworks: one for regulatory decision‐making, and the other for HTA decision‐making. These two frameworks were then compared to examine the potential differences and overlaps in factors between both decision‐making processes.

## 3 Results

The results are presented in three sections: (1) Scoping review on HTA decision‐making, (2) stakeholder interviews on regulatory and HTA decision‐making, and (3) comparisons of factors between regulatory and HTA decision‐making. Here, we summarize the overarching (sub)themes and list identified factors (Tables 1 and 2) for HTA decision‐making, but full descriptions of individual factors, including contributing references to the literature, are reported in Supplementary Material S1. To allow for a comparison between factors related to regulatory and HTA decision‐making, factors found in the previously conducted regulatory‐focused review are also presented in Tables 1 and 2.

**TABLE 1:** Overview of major theme 1, including subthemes and factors, that influence the need or desire for RWE to inform decision‐making.

Theme 1: Questions that can be answered with RWE that facilitate decision‐making					
Regulatory			HTA		
**Subtheme 1.1: Epidemiology**	**No of refs**	**Interviews**	**Subtheme 1.1: Epidemiology and care pathways**	**No of refs**	**Interviews**
**Disease epidemiology (general)**	16 (14%)	+	**Disease and population related aspects**		
Incidence, prevalence, event rates	20 (17%)	+	Incidence, prevalence, event rates	20 (17%)	+
Natural history of a disease	57 (48%)	+	Natural history of a disease	57 (48%)	+
Population characteristics	33 (28%)	+	Transition probabilities between disease states	2 (2%)	+
Landscape of standard of care and treatment patterns	34 (29%)	+	Population characteristics	33 (28%)	+
**Regulatory purposes of epidemiological data**			**Treatment related aspects**		
Contextualisation (general)	3 (3%)	+	Landscape of standard of care and treatment patterns	34 (29%)	+
Contextualisation single arm trial (informal)	67 (57%)	+	Adherence rates	19 (16%)	+
Contextualisation single arm trial (ECA)	21 (18%)	+	Resource utilization	55 (47%)	+
Support orphan designation	1 (1%)	+	Cost of care	42 (36%)	+
Substantiation of single arm trial design	3 (3%)	+			
**Subtheme 1.2: Benefit–risk assessment**			**Subtheme 1.2: Health technology assessment**		
**Pre‐approval benefit–risk**			**Initial reimbursement: REA and economic evaluation**		
Expedited or adaptive approval pathways	26 (22%)	+	Choice of comparators	4 (3%)	+
**Post‐approval benefit‐risk**			Transferability assessment	1 (1%)	+
Continued monitoring of benefit‐risk	37 (31%)	+	Clinical effectiveness	68 (58%)	+
Post approval safety	92 (78%)	+	Safety	92 (78%)	+
Post approval effectiveness	68 (58%)	+	Economic modeling parameters	18 (15%)	+
Conditional approvals	19 (16%)	+	**Alternative reimbursement schemes**		
Evidence gaps related to benefit–risk			Conditional reimbursement schemes	10 (8%)	+
Heterogeneity of treatment effects	34 (29%)	+	Outcomes‐based or pay‐for‐performance schemes	12 (10%)	+
Optimal dosing and frequency of administration	19 (16%)	+	**Other domains and relevant evidence gaps**		
Co‐prescribing	13 (11%)	+	Heterogeneity of treatment effects	34 (29%)	+
Label modifications			Broader impact on the healthcare ecosystem	11 (9%)	+
Population	37 (31%)	+	**Implementation and monitoring**		
Indication	45 (38%)	+	Implementation in clinical practice and quality of care	14 (12%)	+
Other label changes	23 (19%)	+	Monitoring and re‐evaluations	17 (14%)	+
Evaluation of risk minimization measures	14 (12%)	+			

*Note:* Identified themes and factors for regulatory and HTA decision‐making, including the number of references that contributed to each factor (out of a total of 118 articles), and whether they were confirmed (+) in the stakeholder interviews or not (**−**). For a comprehensive description of each individual factor and contributing references, we refer to Supplementary Material S1 (HTA decision‐making) and Supplementary Material S2 of the previously published regulatory review [9]. ECA: external comparator arm; REA: relative effectiveness assessment.

**TABLE 2:** Overview of major theme 2, including subthemes and factors, that influence the need or desire for RWE to inform decision‐making.

Theme 2: Contextual factors that increase the desirability or necessity of RWE in decision‐making		
Regulatory and HTA^a^		
**Subtheme 2.1: Feasibility**	**No of refs**	**Interviews**
Rare populations	75 (64%)	+
Recruitment difficulties	11 (9%)	+
Time constraints	46 (39%)	+
Resource constraints	41 (35%)	+
Long‐term outcomes	68 (58%)	+
Rare outcomes	57 (48%)	+
Multiple comparators and treatment combinations^b^	9 (8%)	+
**Subtheme 2.2: Ethical considerations**		
High unmet need	56 (47%)	+
No equipoise	6 (5%)	+
Vulnerable populations	22 (19%)	+
Other ethical considerations	16 (14%)	**−**
**Subtheme 2.3: Limitations of available evidence**		
Generalizability	16 (14%)	+
Representativeness of endpoint	29 (25%)	+
Representativeness of patient characteristics	80 (68%)	+
Representativeness of patient behavior	19 (16%)	+
Representativeness of treatment setting	24 (20%)	+
Representativeness of treatment protocol	30 (25%)	+
Less robust trial evidence	3 (3%)	**−**
Crossover issues	5 (4%)	**−**
Limited existing knowledge	1 (1%)	+
Absence of head‐to‐head trials^b^	43 (36%)	+
Active comparator not relevant^b^	13 (11%)	+
Relevant outcomes not included in available trials^b^	45 (38%)	+
**Subtheme 2.4: Disease and treatment specific attributes**		
Complex treatment settings	11 (9%)	+
Vaccine research	8 (7%)	+
Changing drug effectiveness over time	3 (3%)	**−**

*Note:* Identified themes and factors for regulatory and HTA decision‐making, including the number of references that contributed to each factor (out of a total of 118 articles), and whether they were confirmed (+) in the stakeholder interviews or not (**−**). For a comprehensive description of each individual factor and contributing references, we refer to Supplementary Material S1 (HTA decision‐making) and Supplementary Material S2 of the previously published regulatory review [9].

^a^ Contextual factors largely overlap between regulatory and HTA decision‐making, and were therefore combined into one column for both decision‐making domains.

^b^ HTA‐specific factors.

### 3.1 Scoping Review: HTA Decision‐Making

#### 3.1.1 Search Results and Article Selection

The search yielded 1435 article hits from electronic databases and 67 from grey literature sources. After removing duplicates, 710 unique articles remained. Screening titles and abstracts led to 217 full‐text reviews, and ultimately, 118 articles met the inclusion criteria and were included in the scoping review. See Figure S2 for selection details.

#### 3.1.2 Synthesis

Two major themes, 6 subthemes and 45 individual factors were identified to influence the need for RWE in HTA decision‐making (see HTA column of Tables 1 and 2). A detailed description of each individual factor, including illustrative quotes and complete reference list, is given in Supplementary Material S1. Table S2 of this document also provides an overview of the references, including their counts, that contributed to each factor. Here, we give a summary of the major themes and subthemes.

##### Theme 1 Questions That Can Be Answered With RWE and Facilitate HTA Decision‐Making

The first major theme describes content‐related questions that could be answered with RWE to facilitate HTA decision‐making.

###### Subtheme 1.1 Epidemiology and Care Pathways

Questions related to characterizing diseases and populations, are often (sometimes inevitably) addressed by RWE, and encompass aspects such as incidence, prevalence, event rates, natural history of a disease, transition probabilities between disease states, and patient demographics. Similarly, RWE proves useful in addressing questions related to treatment characterization. Utilizing electronic health records, prescription data, and claims data, RWE can address questions pertaining to the landscape of standard of care (e.g., current treatment paradigms, thresholds of disease severity at which specific treatments are prescribed, and management of side effects), adherence rates, resources utilization and associated costs (e.g., treatments, diagnostic tests, hospital visits). This information is important to provide clinical context in HTA decision‐making (e.g., to understand the potential placement of a new treatment in clinical practice, interpret the results of traditional trials, etc.), but may also serve as direct input for specific components of the assessment process, as detailed in subtheme 1.2.

###### Subtheme 1.2 Health Technology Assessment

While the specific scope of the HTA may vary across countries, its fundamental components include a relative effectiveness assessment (REA) and an economic evaluation. Therefore, understanding the landscape of standard of care is essential to guide which treatment(s) in clinical practice the new treatment should be compared to. Moreover, RWE (e.g., patient demographics, treatment patterns and resource utilization) is critical to ensure that the evidence that is submitted for HTA, whether originating from traditional trials or other sources, is applicable and transferable to the specific population and setting of interest. The clinical effectiveness component of the REA and economic evaluation is preferably informed by randomized studies (potentially including pragmatic trials generating RWE), although these are often underpowered and too short in duration for the detection of safety signals. Beyond clinical effectiveness and safety, RWE is valuable for informing various parameters and assumptions used in economic models.

RWE can also be utilized for addressing evidence requirements for non‐conventional reimbursement schemes, such as conditional reimbursement schemes and outcomes‐based contracts. Non‐conventional reimbursement schemes differ from traditional reimbursement schemes by tying payment or continued coverage to specific conditions or outcomes. In conditional reimbursement schemes, reimbursement is granted on a provisional basis, subject to further evidence confirming the treatment's cost‐effectiveness. This approach is typically applied to treatments that are promising but have significant uncertainties that need resolution through additional evidence, such as RWE studies. Outcomes‐based contracts may link reimbursement to pre‐defined, individual health outcomes observed in clinical practice, with RWE being essential for tracking these outcomes.

Post‐reimbursement, RWE is useful for evaluating the implementation of the new treatment within clinical practice and measuring the quality of care. Moreover, RWE can be utilized to monitor the actual effects of the new treatment in clinical practice, including real‐world effectiveness, safety and cost‐effectiveness, possibly within patient subgroups. There are also opportunities to evaluate the broader impact of the treatment on the healthcare ecosystem. The insights gained from these post‐reimbursement RWE studies can subsequently be utilized to revisit past decisions and potentially update reimbursement criteria during a continuously evolving standard of care.

##### Theme 2 Contextual Factors That Increase the Necessity or Desirability of RWE in HTA Decision‐Making

The second major theme describes various contextual factors that influence the need for RWE, often stemming from inherent limitations of the traditional trial or the impossibility to conduct one.

###### Subtheme 2.1 Feasibility

The conduct of a traditional RCTs can sometimes be infeasible. In some scenarios, it may be impossible to recruit a sufficient number of participants (e.g., ultra rare diseases or rare patient subgroups). For certain decisions there may be an urgency for immediate evidence, rendering the conduct of a trial impractical. In other situations, the resources required to conduct a traditional trial could become cost‐prohibitive (e.g., rare outcome mandating an exceptionally large sample size). Specifically relevant to HTA decision‐making, if there are multiple comparators (including treatment combinations or sequences) of interest, the execution of a trial incorporating all comparator arms or the pursuit of a series of trials could become too resource intensive and subsequently infeasible. For circumstances where the conduct of traditional trial(s) is infeasible, RWE studies (including RWE to contextualize single arm trials) may provide viable alternative evidence to inform decision‐making.

###### Subtheme 2.2 Ethical Considerations

Ethical considerations may also prevent the conduct of RCTs. For instance, in situations characterized by a high unmet medical need, it may be considered unethical to deny patients access to a potentially efficacious treatment through randomization to a control arm. Likewise, ethical concerns may arise when true equipoise is absent. In these scenarios, a single arm trial might be the only viable option, where RWE could be utilized to contextualize its results.

###### Subtheme 2.3 Limitations of Available Evidence

Certain limitations associated with evidence from traditional trials may underscore the necessity or desirability of incorporating RWE into HTA decision‐making. An often‐criticized aspect of traditional RCTs involves the potentially limited generalizability of their results (e.g., due to strict patient populations included or the use of questionable surrogate endpoints). This issue plays an important role in HTA decision‐making, where submitted evidence should apply to the population, setting and standard of care of the country where the HTA is conducted. RWE can help fill these potential evidence gaps.

###### Subtheme 2.4 Disease and Treatment Specific Attributes

Specific diseases and treatments may further influence the need for RWE in decision‐making. For example, in vaccine research traditional RCTs may face particular challenges, such as non‐serological outcomes that may take a considerable time to develop or difficulties in catching herd effects. The collection of RWE may also be especially important for complex and innovative treatments for which the biological mechanism is not yet well characterized (e.g., “first‐in‐class” products) and long‐term effects are unknown (e.g., gene therapies). For some innovative therapies, learning effects may be present (e.g., cell therapies), where RWE could prove valuable in investigating potential changes in effectiveness over time.

### 3.2 Stakeholder Interviews: Regulatory and HTA Decision‐Making

#### 3.2.1 Participants

A total of 52 stakeholder interview invitations were sent, of which 18 (35%) recipients agreed to participate. Thirty (58%) recipients did not reply (either at all, or to follow‐up emails to set an appointment), and 3 (6%) recipients actively declined. All three recipients who declined mentioned their employment at a regulatory agency (EMA, FDA) as a barrier to respond to the interview questions on a personal title. Participants' stakeholder roles as well as their geographical perspectives, are outlined in Table 3. A substantial part of the participants (10/18, 56%) were active or had extensive experience within multiple stakeholder groups. Although not listed as a separate stakeholder group here, two participants were working as statisticians.

**TABLE 3:** Characteristics of participants in stakeholder interviews on the need for RWE in regulatory and HTA decision‐making.

	Geographical perspective	
Stakeholder roles	Europe^a^	North‐America^b^
Total participants	12	6
**Single roles**		
Regulator	2	
Academia	1	
Patient advocate	2	
Pharmaceutical industry	1	
Data provider	1	
Consultancy and data analytics provider		1
**Multiple roles**		
Regulator, HTA	1	
Regulator, HTA, academia	1	
Regulator, clinician	1	
HTA, academia	1	
Academia, consultancy and data analytics provider		2
Academia, data provider		1
Data provider, pharmaceutical industry		2
Patient advocate, data provider	1	

^a^ Denmark, France, Netherlands, Norway, Switzerland, United Kingdom.

^b^ United States of America.

#### 3.2.2 Synthesis

Sixty‐three factors were identified in the stakeholder interviews, which aligned with factors and themes previously identified in the scoping reviews on regulatory and HTA decision‐making (Tables 1 and 2). A sample of quotes by participants supporting themes and factors are listed in Table 4. Additionally, participants discussed several barriers for RWE in the context of decision‐making processes (Box 1).

**TABLE 4:** Quotes from participants in the stakeholder interviews, that support (sub)themes influencing the need for RWE in decision‐making (next page).

Themes, subthemes and factors	Quotes from stakeholder interviews	
**Theme: Regulatory questions that can be answered with RWE and facilitate decision‐making**		
**Subtheme 1: Epidemiology**		“Whether the medicine is to have the orphan medicinal product status, that's a decision made by the Committee for Orphan Medicinal Products, and they decide if the product can receive financial incentives or scientific incentives. So it's regulatory decision‐making. And here already RWD are needed, for example, just to know how many patients have the disease in question.” “We also think about doing descriptive studies to characterize the natural history of disease. And what that means to me is that you are characterizing the risks of outcomes in the indicated population under whatever treatments are available currently, including no treatment at all, trying to understand, what kinds of patients progress through different stages of disease.” “Other ways that descriptive studies can be very useful, is looking at background rates of potential safety issues in the indicated population. I've seen this useful when in a phase three trial, there was an imbalance of an unexpected rare safety event.” “Sometimes you're left with single arm data, but in order to give some context, you use RWE to get the background information on the untreated population. And you can do that more formally with an external comparator arm.” “We are also doing a lot of work for regulatory agencies, including natural history and descriptive epidemiology, just to help them understand the condition or the utilization of drugs. And some of the information is being used to consider a review or approval of a new product.” “A typical question at scientific advice when discussing even an RCT is, which is the most relevant comparator? […] Already at this stage, drug utilization studies, which are RWD studies, are needed to have an idea on all treatments used for the disease and the frequency of their use, to have an informed discussion on what is the best standard of care to use as a comparator.”
Disease epidemiology	+	
Incidence, prevalence, event rates	+	
Natural history of a disease	+	
Population characteristics	+	
Landscape of standard of care and treatment patterns	+	
Regulatory purposes of epidemiological data		
Contextualisation (general)	+	
Contextualisation single arm trial (informal)	+	
Contextualisation single arm trial (ECA)	+	
Support orphan designation	+	
Substantiation of trial design	+	
**Subtheme 2: Benefit–risk assessment**		“It can help support a case for an expedited program designation. In the US, we have designations for fast track, breakthrough—these kinds of designations are reflective of an urgent public health need coupled with some early evidence of benefit, so that FDA's review times become more compressed in response to those considerations. And without good natural history data, you can't make those arguments.” “Post‐approval safety is a critical use and frankly the most historic use of RWD and observational methods.” “RWD could be used for post‐marketing commitments and requirements. And those kinds of opportunities, in many ways are coming up for marketing applications where you have conditional approvals or accelerated approvals.” “Once you have proof from an RCT, you know that in this group, you saw this effect. But in the real world, there's lots of different types of people, and they respond differently to treatments. So the more you monitor different groups of patients, you have a possibility to see how they respond differently to this treatment.” “Especially as you then expand into populations with different profiles of co‐morbidity, of socio‐demographic status, of polypharmacy situations, we may not know about. That's where I will argue that, if not already then definitely over the years to come, RWE will be necessary.” “The Pediatric Research Equity Act mandates that unless there is some argument to be made about why the indication is not applicable to children, that sponsors must provide evidence of effectiveness and safety in children as a post‐approval requirement. And I've been involved in several examples where that evidence was provided by RWE. And then that has led to a label change where the labeling then explicitly calls into scope pediatric patients.” “We've had examples of a product where risk minimization measures could be implemented during the compassionate use program, and be evaluated demonstrating they were effective at reducing the risk before the end of the evaluation of the product. So regulators had a real‐life demonstration of the efficacy of risk minimization measures before they decided to finally authorize the product.”
Preapproval benefit–risk		
Expedited or alternative approval pathways	+	
Post approval benefit–risk		
Continued monitoring of benefit–risk	+	
Post approval safety	+	
Post approval effectiveness	+	
Conditional approvals	+	
Evidence gaps related to benefit–risk		
Heterogeneity of treatment effects	+	
Optimal dosing and frequency of administration	+	
Co‐prescribing	+	
Label modifications		
Population	+	
Indication	+	
Other label changes	+	
Evaluation of risk minimization measures	+	
**Theme: HTA‐relevant questions that can be answered with RWE and facilitate decision‐making**		
**Subtheme 1: Epidemiology and care pathways**		“All of that information (characterization of unmet need, patient population, treatment pathways and standards of care) will be useful to an HTA to give contextual considerations of this new drug entering the market. So all of that background information is absolutely necessary for an HTA to understand how this drug or product is going to come in and potentially impact the treatment pathway, impact patient care, and so forth.” “An awful lot of work that we are asked to do and commissioned to do at the moment is around understanding that landscape for the current treatment pathways, the current healthcare resource utilization, to get a good picture of the status quo.” “A large group of HTAs in Europe do cost‐utility analysis. […] Let's say at the first screening, I have to make a decision whether the patient is likely going to have progression or not. That comes from the clinical trial data, for example. But then the longer we go, the less likely do we have clinical trial data. Hence, I need this information from somewhere else. So I'll take the likelihood of such a patient from a RWD source.” “I think in addition to contextualizing the patient population, you also need to understand the costs and the cost savings that this new entry might bring. So for example, the cost of the drug is probably going to be high, but if it avoids hospitalizations, which are very costly, on the bottom line, that's important to an HTA to know.”
Disease and population related aspects	+	
Incidence, prevalence, event rates	+	
Natural history of a disease	+	
Transition probabilities between disease states	+	
Population characteristics	+	
Treatment related aspects		
Landscape of standard of care and treatment patterns	+	
Adherence rates	+	
Resource utilization	+	
Cost of care	+	
**Subtheme 2: Health technology assessment**		“It's the reason why some early interactions are being organized with the HTA bodies to determine with them if the comparator arm would be the good one in accordance with treatment guidelines, cost effectiveness, and so on. […] So I would say that we have treatment guidelines which are guiding us regarding what the comparator should be. And when we interact with HTA we also use RWE in our discussions.” “But I even think that in the case of having clinical trial evidence, information on how resources are utilized, in what ways, and for which populations, is relevant. It helps you interpret clinical trial evidence, and say ‘Yes, that is applicable to the population being treated in our country, or not.’ “ “So in addition to understanding of unmet need, understanding of the patient population, I think costs, adherence, and other inputs to a cost‐effectiveness model are important to gather in the clinical development phase.” “Accelerated approvals to getting the patients these drugs has increased. It decreases the potential clinical evidence at launch, which increases the uncertainty. And in response to that increased uncertainty, we're seeing HTA agencies have different mechanisms in place, like coverage with evidence development or managed access agreements, where they are asking the sponsor to resubmit additional evidence within a particular timeframe, and some of that evidence can be RWE.” “Obviously, there's opportunity to use RWE to set up those outcomes‐based contracts, understand what patient population should be included, what outcomes are relevant, how to measure those outcomes, when to measure those outcomes.” “[…] the real‐world element of that looking at things over a longer time scale is important not only for those original decisions, but looking at things on an ongoing basis over time.” “And we've seen things that were not even measured during the clinical trials, in terms, for example of women with cystic fibrosis who can now have babies. And that wasn't the case before. They would not take the risk of becoming pregnant. Now the efficacy of the treatment is such that hundreds of women in Europe with cystic fibrosis gave birth to babies. And in itself, it's a huge progress for them of course, but it's also an indication that there is so much information that can be collected after development to inform on the societal utility value of the treatment.” “That's an aspect that I actually don't hear much about in the discussions around healthcare reimbursement, patients' ability to work and participate in society and such. I find it an underemphasized aspect, and I hope that RWE will provide us with more insights on this.” “You could even argue the societal impact of absenteeism, for example. You could argue that is important, and could be collected with RWD.”
Initial reimbursement: REA and economic evaluation		
Choice of comparators	+	
Transferability assessment	+	
Clinical effectiveness	+	
Safety	+	
Economic modeling parameters	+	
Alternative reimbursement schemes		
Conditional reimbursement schemes	+	
Outcomes‐based or pay‐for‐performance schemes	+	
Implementation and monitoring		
Implementation in clinical practice and quality of care	+	
Monitoring and re‐evaluations	+	
Other domains and relevant evidence gaps		
Heterogeneity of treatment effects	+	
Broader impact on the healthcare ecosystem	+	
**Theme: Contextual factors that increase the desirability or necessity of RWE in regulatory and HTA decision‐making**		
**Subtheme 1: Feasibility**		“Well, 90% of the argument is there are just not enough patients around. You have these ultra rare diseases, you have the rare diseases, you have the highly targeted treatments and other conditions where you have biomarkers to identify the patients of interest. […] Then it just gets very hard to recruit subjects into randomized trials and then you don't want to move subjects into the comparison arm. You want to preserve them all in the exposed to learn about exposure and treatment response and things like that.” “Especially when it is difficult to recruit patients. In oncology, I would say the landscape is very competitive. You can have maybe 40 clinical trials with the same comparator arm. So, we are discussing internally the possibility to propose some hybrid control arm with RWD.” “The other obvious thing that you often lack from the pre‐approval trials is enough safety insights, because these trials are limited in size and that's of course where all the PASS studies and post‐marketing requirements come in. So for those rare outcomes and for long‐term outcomes, you would want to use RWE. “ “One is cost. We know the expense of an RCT, how many millions of dollars it is.” “We are increasingly dealing with various treatment combinations, which could also be used in various sequences. You really can't study all of that in the form of RCTs. So you will need data from clinical practice to form a better understanding.”
Rare populations	+	
Recruitment difficulties	+	
Time constraints	+	
Resource constraints	+	
Long‐term outcomes	+	
Rare outcomes	+	
Multiple comparators and treatment combinations^a^	+	
**Subtheme 2: Ethical considerations**		“A recent example was for CAR T‐cell therapy, where I think the disease was one of the lymphomas, where there was an estimated 3000 patients in the EU. So in theory, enough patients to randomize in a clinical trial, compared to standard of care, but with a devastating disease, where standard of care was known to be poorly effective. […] A priori data from the early development indicated such a high effect of the medicine that in this context, no patients would have accepted to be randomized, and no clinician would have accepted a design which would not have been an open‐label trial.” “We see it sometimes with advanced therapies where animal data, preclinical data, indicate that the effect of the treatment can be so high that, the question of equipoise can make clinical trials impossible to design.” “I think there's obviously certain groups where it's just not ethical to conduct traditional randomized controlled trials. A lot of the focus over the last few years has been on safety of medicines in pregnancy and rightly so.”
High unmet need	+	
No equipoise	+	
Vulnerable populations	+	
Other ethical considerations	**−**	
**Subtheme 3: Limitations of available evidence**		“And that is where you reach this problem of RCTs. They are restrictive. They are not the real world.” “To me, randomized clinical trials is not real life. Because, as you know, you have a list of inclusion, exclusion criteria. So, the population can be a little bit different from the real‐world. In addition, you have regular visits with regular intervals and so on. And it is done according to a protocol. So, you can have some gaps between the efficacy data you have from randomized clinical trials and effectiveness data you may have in the real‐world context.” “What are the other benefits that I can derive using a pragmatic study or a point of care study and that I don't get from a traditional RCT? It's access to underrepresented population. I can't speak for Europe, but for the United States, it's having access to patients in community centers that are not in major academic centers. So having a different swath of patients, not just from under‐representation, but for different types of clinical practices that aren't typically represented in clinical trials because clinical trials are traditionally in large medical centers. That's where they're conducted. And the type of care that patients get there is not the care that they get in the rest of the country.” “There is indeed a difference between a disease that you know inside out, with clear guidelines and a large patient population, where you can much better assess whether an RCT is well‐designed and has good predictive value. However, with smaller, less‐known disease areas or unclear patient populations, the judgment is often much more challenging. Does this RCT really examine the right things? So as uncertainty increases, I believe you also want to make the totality of the evidence you have available as large as possible, and also really engage with patient groups to get a better understanding.” “A frequent problem with data from RCTs is that they either have used a placebo as a comparator, or a treatment that is not relevant for the Netherlands.” “It's very difficult because you can choose a comparator, and then a new approval is obtained by a competitor and then you are in a difficult situation.” “In HTA organizations, they're always looking at the relative effect of a particular product compared to the standard of care. And usually that standard of care is not what was used in the randomized control trial. Either the randomized control trial used placebo or the country has very different standards of care that wasn't necessarily used as the comparator in the RCT.”
Generalizability	+	
Representativeness of endpoint	+	
Representativeness of patient characteristics	+	
Representativeness of patient behavior	+	
Representativeness of treatment setting	+	
Representativeness of treatment protocol	+	
Less robust trial evidence	**−**	
Crossover issues	**−**	
Limited existing knowledge	+	
Absence of head‐to‐head trials^a^	+	
Active comparator not relevant^a^	+	
Relevant outcomes not included in available trials^a^	+	
**Subtheme 4: Disease and treatment specific attributes**		“I think that the costs of orphan drugs are getting out of hand. They are asking for way too high prices. So, I believe that the solidarity of our healthcare system is at stake. We need to better demonstrate the value of therapy for the patient. Because there is certainly a need, but we also need to show what the drug does and its real value. In the real world, with real patients. Especially with things like AAV gene therapy, of which we don't know exactly how long it works.” “I think, for example, of the entire group of psychiatric conditions. There, you often deal with patient‐reported outcomes, influences of other (non‐pharmaceutical) therapies on the outcome. They are often much more challenging to make a good benefit–risk assessment. Wouldn't you want to have a much richer database with data for such conditions, where RWD really have a place? […] Now you often find yourself in the context of evaluating an RCT, and you have to work with rather meager data on softer endpoints. The conclusion is often ‘Well, it seems to do something, but is that really the case?’ Well, it's difficult to pass a firm judgment on that. In my opinion, you would prefer to have a much larger source of data and much more insight into what exactly is that disease, what are the exact characteristics, so that you have a stronger foundation for evaluating a specific dossier.” “…especially around the thrombo‐embolisms with the vector‐based vaccines that led to the discontinuation of those vaccines in a Danish and Norwegian setting. So obviously we needed to provide them a second shot for the boosting of the immunization, leading to the use of the mRNA vaccine as a second shot. That would be considered an off‐label use, because no one had actually seen or been granted a market authorization, nor had that been provided with necessary evidence to be a normal treatment option. So we did real‐time surveillance using RWD, creating the evidence for us that this was actually safe to do.” “If you have these heterologous vaccines that you have been administered, would that have a different effect than the homologous vaccines that we would be administering? […] So across the board for both understanding disease progression and the use of existing drugs, for monitoring of new vaccines, but also to react on potential adverse drug reactions, and then obviously then in collaboration efforts to really understand also effectiveness of vaccines in the Nordic settings.”
Complex treatment settings	+	
Vaccine research	+	
Changing effectiveness over time	**−**	

*Note:* For a comprehensive description of each individual factor, we refer to Supplementary Material S1 (HTA decision‐making) and Supplementary Material S2 of the previously published regulatory review [9]. + factor identified from literature was also mentioned and confirmed by participants in stakeholder interviews. − factor identified from literature was not mentioned by participants in stakeholder interviews.

^a^ HTA‐specific factor.

BOX 1 **Potential barriers mentioned by participants to the use of RWE in decision‐making processes.**

During stakeholder interviews, participants spontaneously expressed various barriers to the use of RWE in decision‐making processes, of which several prominent topics are outlined below.

**RWD related barriers**

- Relevant outcomes for decision‐making are often not captured in real‐world data (RWD) sources
- Missing data
- Misregistration and ‐classification
- Lag time
- Governance
- Heterogeneity in RWD sources (e.g., due to differences in healthcare systems) can limit the possibility to combine datasets and perform larger scale, international RWE studies.
- Ethical considerations in commercial use of citizen RWD by pharmaceutical companies
- Potential in using placebo arms from prior trials as external comparator arms (ECAs), but data rarely available

*“[…] and if the data contain all the elements that you require to answer a question in a certain context. That is rarely ever the case […] There's a complete misalignment between the ominous availability of RWD, and moving RWD into RWE in a relevant context.”*

*“We need to make sure that also the clinicians understand why it is so important that they register every piece of data correctly […] That is another whole area of RWE that is so poorly explored.”*

*“I think it's getting better and better with the years, but it's still often insufficient; the data granularity, the data completeness, and also the lag time that we see in data generation.”*

*“[…] because of governance at the moment, it's just too difficult on a large scale to link together pupil data with health data. And that's a barrier to answering some really important societal questions.”*

**Barriers mentioned in relation to regulatory decision‐making**

- Lack of a formal decision‐making framework that includes RWE may negatively impact consistency in regulatory decisions, and prevents clarity on its role and necessity

*“That's the part where the regulatory system is very opaque. So while the HTAs have a very structured way of including RWE, the CHMP [Committee for Medicinal Products for Human Use] doesn't. […] The discussion ends up often lacking consistency simply because there is no guideline on how to handle RWE provided by an applicant in the benefit‐risk assessment, the EPAR [European public assessment report], and the decision‐making of the approval […] And unless you have a formal framework, it'll remain your gut feeling, me right, you wrong kind of approaches, which are not good because that doesn't strengthen the role and it doesn't strengthen the need for it.”*

- There is an increasing interest in pragmatic trials (e.g., for fulfilling conditional evidence requirements or label expansions), but they are not often performed

*“There's lots of interest from academia, data providers and service providers going, “look, we could do this.” But for pharma to take the risk, to put money into it, it's like, well, where are your examples that regulators have accepted this [evidence from pragmatic trials] for key decision making? And that's difficult, because we're just not there yet, really.”*

*“In the real world, you can do randomization, people keep forgetting about this, they don't really understand it, you can run a randomized experiment in a RWD source.”*

*“It seems, just in my experience, it's a little bit harder to lift some of those [pragmatic trial] designs off the ground.”*

**Barriers mentioned in relation to HTA decision‐making**

- Delays in RWE needed for HTA decision‐making in Europe (e.g., timely RWD regarding disease state transition probabilities in standard of care, to facilitate extrapolations of trial data beyond trial durations, as input for economic models)

*“That is our biggest problem. If drug developers would in phase 2A‐B start talking about what do we need for the HTAs rather than only for the CHMP, they would understand that they should, for example, run maybe a study in a registry to allow generating this RWE concurrently with their ongoing studies. […] Fixing it afterwards in post‐authorization takes years and frustrates everyone.”*

*“It is on the drug developer's mind very much so, that there is the HTA hurdle, and they know what they like to see, yet they sometimes still make decisions that they will focus on the medicine's regulator first and provide the evidence very targeted for that decision‐making and sacrifice the evidence generation for the follow‐up decision. And here comes the cynical part that many pharma companies don't see Europe as the primary market anymore. They say, “Look, the HTA is just a pain in the neck, I don't want to deal with them. I market in the US, that's my primary market, and as long as I get market access there, I will deal with the HTAs later on. I will generate more evidence later on,” which is why increasingly we see the evidence needs for HTA is not satisfied at the time of approval, but comes later and later.”*

*“There is a new legislation regarding HTA in Europe, where ultimately assessments must be done collectively. The aim is also to provide early advice to the manufacturer on what needs to be collected collectively, and even in parallel with EMA. The idea is that if Europe acts more as a united front and says, “This is what we need,” it will have a greater impact on what the manufacturer does.”*

- Difficulties in non‐conventional reimbursement schemes and re‐evaluations

*“There are certain realities that is driving why we see way fewer value based coverage arrangements than should be. One thing is that, […] you have to formulate exactly your metrics in lawyer English. And that often results in stark simplification of the evidence generation […] The other thing in the US is you have the Pharmacy Benefit Managers in the middle that destroy any value conversation because they just sell by volume.”*

*“The issue is always who is allowed to actually decide success or failure, based on what?”*

*“At least in the US, I think they're disincentivized to do more complicated outcomes‐based agreements when they can just kind of negotiate a bigger discount, as opposed to jumping through all the hoops of an outcomes‐based contract.”*

*“There's a lot of talk around health technology management and reassessing products, but other than those very high cost products with a lot of uncertainty, we're not seeing too much of a shift toward continual reassessments on a particular timeframe.”*

#### 3.2.3 Confirmation of Factors Identified From the Literature

All of the views, concepts and use‐cases mentioned by participants that could increase the necessity or desirability of RWE to inform regulatory and HTA decision‐making, aligned with factors previously identified in the scoping reviews, and thus could be categorized using existing themes and factors. In total, 67 factors were identified from the literature (regulatory and HTA scoping review), of which 63 (94%) were mentioned by participants. The four factors that were not mentioned by participants, included other ethical considerations (e.g., patients unwilling to be allocated to one of the intervention arms if another treatment is perceived as the most optimal one, even if scientifically unproven), less robust trial evidence, crossover issues, and changing effectiveness of a treatment over time (i.e., learning effects). While no new factors or themes increasing the need or desire for RWE to inform regulatory and HTA decision‐making were identified from the interviews, participants did provide more in‐depth information about use cases for certain factors, as illustrated by some of the quotes in Table 4.

### 3.3 Comparisons Between Regulatory and HTA Decision‐Making

#### 3.3.1 Similarities in Question Factors Between Regulatory and HTA Decision‐Making

In both the regulatory and HTA domain, RWE regarding disease, population, and treatment aspects is valuable to provide context. This information helps with the interpretation of traditional trial results, can be used to contextualize single arm trials, and is useful during scientific advisory meetings with decision‐makers. RWE also aids in the assessment whether submitted evidence is applicable and transferable to the setting and population of interest. After market entry, RWE assumes a pivotal role in the monitoring of long‐term safety and effectiveness of treatments in real‐world settings. Additionally, it presents opportunities to investigate other potential evidence gaps pre‐approval trials may have been unable to address, such as heterogeneity of treatment effects. While these applications of RWE are relevant to both regulatory authorities and HTA decision‐makers, regulatory authorities may have a more established role in requesting and enacting upon post‐approval data collection (particularly in addressing safety signals and employment of risk minimization measures, although market retractions are rare). Revisiting and updating past reimbursement recommendations based on post‐market entry RWE remains relatively limited in the HTA setting. Moreover, parallels can be drawn between conditional regulatory approvals and conditional reimbursement schemes, both of which may benefit from addressing conditional evidence requirements through utilization of RWE.

#### 3.3.2 Differences in Question Factors Between Regulatory and HTA Decision‐Making

In conjunction with the shared purposes of RWE between the regulatory and HTA domains, there are also differences. Regulators additionally require RWE (e.g., incidence, prevalence and burden of disease) to inform decision‐making regarding submissions for orphan status and alternative approval pathways. In HTA decision‐making, RWE guides the selection of appropriate comparators for the new treatment, and is necessary to populate and inform assumptions in economic models. In contrast to the benefit–risk assessment in regulatory decision‐making, RWE thus also serves as direct input for the HTA, and involves specific details (e.g., transition probabilities between disease states, adherence rates), and a broader scope than what is required for regulatory decisions (e.g., costs of resource utilization). Additional differences between the questions addressable with RWE in HTA decision‐making, that are typically beyond the scope of regulatory considerations, encompass the effective implementation of treatments in practice, the quality of care delivered, and the broader implications of a treatment on the healthcare system (e.g., absenteeism, health inequalities, changes in disease prevalence and transmission rates after introduction of a vaccine). Conversely, RWE can be utilized to evaluate the effect of risk minimization measures, a facet unique to regulatory decision‐making.

#### 3.3.3 Contextual Factors Influencing the Need for RWE in Regulatory and HTA Decision‐Making

Notably, the contextual factors influencing the need for RWE in regulatory and HTA decision‐making exhibit considerable overlap. Most contextual factors that increase the need or desire for RWE are tied to circumstances where RCTs are not possible to conduct, or have inherent limitations. Given that evidence derived from RCTs forms the cornerstone for both the benefit–risk assessment and the effectiveness and safety components of the HTA, the factors logically intersect between regulatory and HTA decision‐making.

However, within the HTA context, certain additional factors emerge that may further increase the need for RWE. As HTA decision‐makers consider relative effectiveness and a broader set of outcomes, traditional trials that are unable to deliver on those aspects (e.g., omission of patient‐relevant outcomes, absence of active comparators) could subsequently increase the need for additional RWE. In tandem with this, the potential issue of limited generalizability of traditional trial evidence may be more impactful in HTA decision‐making. While the consideration of generalizability and applicability of trial evidence holds importance in regulatory decision‐making (e.g., patient demographics), its significance is likely greater in HTA context, where reimbursement decisions are typically made on a national basis, and involve relative cost‐effectiveness predictions tailored to a real‐world, country specific population and setting.

## 4 Discussion

Building on the findings of our previous scoping review regarding the need for RWE in regulatory decision‐making, in the present study, we identified factors reported in literature that influence the need or desire for RWE in HTA decision‐making. In addition, we confirmed these factors by means of stakeholder interviews for both regulatory and HTA decision‐making, and evaluated how factors overlap and differ between regulatory and HTA decision‐making.

Based on the literature, 45 factors were identified that influence the need for RWE in HTA decision‐making. The first theme described the questions addressable with RWE that facilitate HTA decision‐making, with subthemes epidemiology and care pathways, and health technology assessment. The second theme included contextual factors, with subthemes feasibility, ethical considerations, limitations of available evidence, and disease and treatment specific aspects. Together with the findings from our previous scoping review covering the regulatory domain, a total of 67 factors were found that influence the need or desire for RWE in regulatory and/or HTA decision‐making [9]. The vast majority (63/67; 94%) of these factors was confirmed in the stakeholder interviews. Although no new factors were identified in the stakeholder interviews, the interviews provided more in‐depth examples of when RWE could be valuable to inform decision‐making, as well as several barriers to the use of RWE.

When looking at potential similarities and differences between the factors in regulatory and HTA decision‐making, a few things are evident. While there are parallels in the questions that are addressable with RWE for regulatory and HTA decision‐making, there also substantial differences, to a large extent due to the broader scope of the HTA (e.g., costs, relative effectiveness) while also requiring specific details (e.g., transition probabilities between disease states, adherence rates), that serve as direct input for the HTA. These differences likely lead to an overall increase in the need for RWE to inform HTA decision‐making. In contrast, the contextual factors between the regulatory and HTA domain are predominantly the same, with only the addition of a few factors for HTA (which also relate to its broader scope, e.g., absence of head‐to‐head trials, non‐relevant active comparator). However, it is noteworthy that the contextual factors relating to the potentially limited generalizability of trial evidence, are likely more impactful in HTA decision‐making.

The parallels between the role of RWE for regulatory and HTA decision‐making could help increase efficiency in evidence generation processes during drug development. A single, well‐planned RWE study, has the potential to serve various purposes within regulatory and HTA decision‐making processes. For example, RWE on population and treatment aspects can be useful for scientific advice with regulators and HTA decision‐makers, orphan status submissions or alternative approval pathways, provide clinical context for the interpretation of trial results, as well as an assessment of their transferability, and informing comparators and economic model parameters for the technology assessment. Similarly, if certain contextual factors are present (e.g., a rare patient population with a high unmet need, rendering a traditional RCT infeasible) the assessment of benefit and harms, necessary for both regulatory and HTA decision‐making, may benefit from insights derived from RWE. The EMA‐HTA joint clinical assessments provide an interesting platform where, in the case of conditionally approved medicines, a carefully designed post‐authorization RWE study could potentially satisfy evidentiary needs for both full regulatory approval and the technology assessment [14]. This approach could streamline the process by aligning the requirements for full approval with those for HTA evaluation, thereby reducing the need for multiple separate studies.

This overview of factors may be helpful in recognizing circumstances where RWE might address evidentiary needs of decision‐makers, potentially preventing duplicate efforts and potential unnecessary delays in patient access later on (e.g., during reimbursement decisions). Our findings may be useful to sponsors during early drug development, as well as for early dialogues, joint scientific advisory meetings, and joint clinical assessments with regulators and health technology assessors. In addition, it could contribute to an overall increased mutual awareness between decision‐makers to facilitate convergence of evidentiary needs [15]. Greater awareness of the factors that may be considered by different parties could prevent miscommunication between parties and accelerate drug access. What is more, the list of factors could be considered a comprehensive starting point in assessing the value of RWE (i.e., what weight should be attributed to RWE in the decision‐making process).

For RWE to serve evidentiary needs of both regulators and HTA decision‐makers, alignment of outcomes and study designs may be required, or, if not possible or preferred, RWE studies should be sufficiently inclusive (i.e., covering necessary outcomes to facilitate both regulatory and HTA decision‐making processes) [15, 16]. Furthermore, it is important to recognize that the persuasiveness and weight of RWE in decision‐making, not just depends on the need for RWE, but also on other critical factors, such as data quality, methodological quality and the consequences of the decision to be made. This could potentially lead to differences in the eventual impact of RWE in regulatory and HTA decision‐making, even if the need for RWE between both decision‐makers is shared.

### 4.1 Strengths and Limitations

We used two complementary approaches to identify factors influencing the need or desire for RWE in regulatory and HTA decision‐making: namely, scoping reviews of the literature, and stakeholder interviews. While the reviews summarized extensive amounts of information, there is a risk that they may not be up‐to‐date, as discussions on RWE continue to evolve, and it takes time for new perspectives to be published. Additionally, we found that certain stakeholder roles were underrepresented in the literature (e.g., almost no patient advocate authors were present in our sample of articles) [9]. The stakeholder interviews should have addressed some of these gaps by offering a more recent view on the need for RWE, drawn from a broad and more balanced mix of stakeholders (including regulators, health technology assessors, pharmaceutical industry, data providers, data technology providers, academia, clinicians and patient advocates). These complementary approaches should increase the comprehensiveness of our results. However, several limitations apply. The number of interview participants was limited, and participants were recruited from the author's personal network. Moreover, the interview topic (RWE to inform decision‐making) was explicitly mentioned in invitation emails. This may have resulted in biased participation (e.g., inclusion of participants with a more favorable view toward RWE), although all intended stakeholder groups were represented in the interviews. Furthermore, interviewees represented only a limited number of countries (restricted to Western Europe and United States), and certain stakeholder role‐geographical perspective combinations were lacking (e.g., regulators from a North‐American context). This may have resulted in a more selective range of opinions in the interviews. Since the literature review focused on decision‐making in Europe and North‐America, results may similarly not apply to other regions. Finally, coding and analysis was performed by one author, and qualitative analyses can be subject to personal interpretation. However, identified factors and themes were reviewed and discussed within the research team, and subsequently refined to increase the consistency of interpretation.

## 5 Conclusion

The contextual factors driving the need for RWE are similar between regulatory and HTA decision‐making, and often relate to scenarios where RCTs are insufficient or infeasible. However, the questions addressable with RWE that facilitate decision‐making partly differ between the regulatory and HTA domain. In both domains, RWE provides essential context to interpret trial results, assess applicability and transferability of evidence, and help fill evidence gaps that RCTs may not address, such as long‐term outcomes and heterogeneity of treatment effects. However, where regulators focus primarily on benefit–risk assessments, HTA decision‐makers consider comparative (cost‐) effectiveness and broader healthcare impacts. These broader healthcare impacts, as well as certain components of comparative cost‐effectiveness assessments, are directly informed by RWE (e.g., costs, adherence rates, disease states transition probabilities), in contrast to the benefit–risk assessment. Conversely, regulators use RWE to inform decision‐making surrounding orphan designation and alternative approval submissions, as well as to evaluate the effect of risk minimization measures. The current overview of factors may help sponsors and other stakeholders recognize opportunities where RWE generation processes can be optimized, and serve evidentiary needs of both regulators and HTA decision‐makers.

### 5.1 Plain Language Summary

Real‐world evidence (RWE) is increasingly considered in drug approval and reimbursement decisions, alongside the evidence from randomized controlled trials (RCTs). Yet, the need for RWE may differ between these decisions. Recently, a literature review was conducted that investigated factors that influence the need for RWE in drug approval decisions. In the current study, we used this review as a basis to now investigate the need for RWE in reimbursement decisions, after which interviews with stakeholders were held to confirm and enrich factors from both reviews. Factors were then compared between drug approval and reimbursement decisions. For reimbursement decisions, the need for RWE was found to depend on the questions that need to be answered in order to facilitate decision‐making, and various contextual factors that are related to feasibility and ethical considerations of RCTs. Limitations of available evidence, and disease and treatment specific aspects also contribute to the need for RWE. These contextual factors were largely the same for drug approval decisions, but question‐related factors partly differed. Reimbursement decisions consider broader aspects, such as healthcare costs, that RWE can address, while also more often requiring evidence tailored to specific populations and healthcare systems than what is generally needed for drug approval decisions.

## Conflicts of Interest

The authors declare no conflicts of interest.

## Supporting information


**Figure S1.** Simplified schematic of a medicine's lifecycle.

**Figure S2.** PRISMA flow diagram of the article selection process.

**Table S1.** Scheme of the interview guide.

**Supplementary Material S1.** List of factors that increase the desirability or neccessity of RWE in HTA decision‐making.
