# Supplementary material for: Multiple Perspectives on the Need for Real‐World Evidence to Inform Regulatory and Health Technology Assessment Decision‐Making: Scoping Review and Stakeholder Interviews
Source: Pharmacoepidemiol Drug Saf. 2025 Jan 7;34(1):e70074. doi: 10.1002/pds.70074 (PMC11706668; doi:10.1002/pds.70074)
Supplement: Supplementary file 1 — Figure S1. Simplified schematic of a medicine's lifecycle. [file PDS-34-e70074-s003.docx]

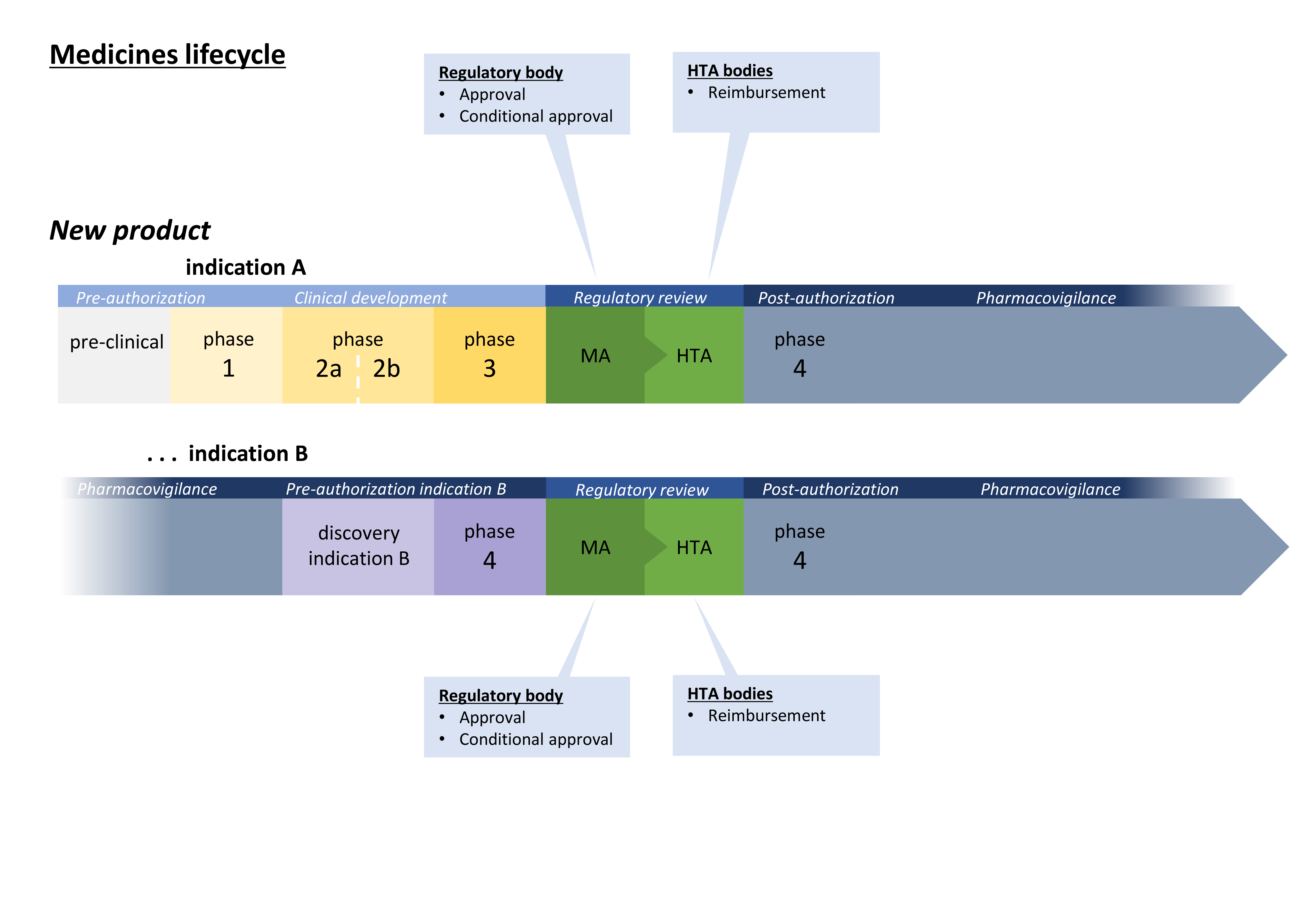


**Figure S1. Simplified schematic of a medicine’s lifecycle**

This slide was used during stakeholder interviews, to help elicit views and ideas regarding the need for RWE in regulatory and HTA

decision-making, as well as discuss the potentially varying need of RWE throughout a medicine’s lifecycle.
*RWE: real-world evidence; MA: market approval; HTA: health technology assessment*
