# Supplementary material for: Multiple Perspectives on the Need for Real‐World Evidence to Inform Regulatory and Health Technology Assessment Decision‐Making: Scoping Review and Stakeholder Interviews
Source: Pharmacoepidemiol Drug Saf. 2025 Jan 7;34(1):e70074. doi: 10.1002/pds.70074 (PMC11706668; doi:10.1002/pds.70074)
Supplement: Supplementary file 2 — Figure S2. PRISMA flow diagram of the article selection process. [file PDS-34-e70074-s001.docx]

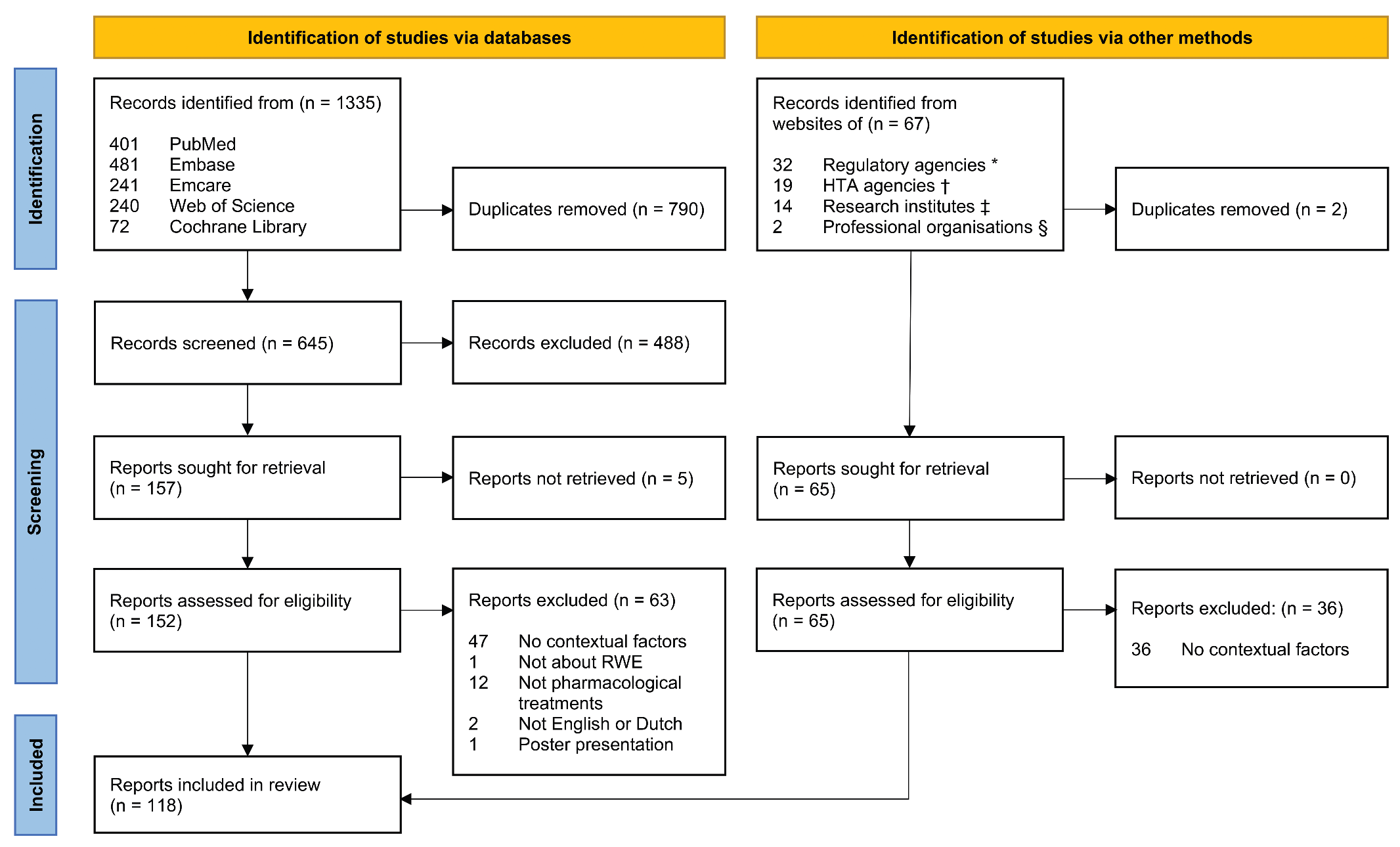


**Figure S2. PRISMA flow diagram of the article selection process*** EMA, MHRA, FDA, Health Canada
† EUnetHTA, NICE, ZIN, ICER, CADTH
‡ Duke-Margolis, ImpactHTA, GetReal Institute
§ ISPE, ISPOR, HTAi, INAHTA
