# Supplementary material for: Multiple Perspectives on the Need for Real‐World Evidence to Inform Regulatory and Health Technology Assessment Decision‐Making: Scoping Review and Stakeholder Interviews
Source: Pharmacoepidemiol Drug Saf. 2025 Jan 7;34(1):e70074. doi: 10.1002/pds.70074 (PMC11706668; doi:10.1002/pds.70074)
Supplement: Supplementary file 3 — Table S1. Scheme of the interview guide. [file PDS-34-e70074-s002.docx]

**Table S1 Scheme of the interview guide**

| **Part** | **Topic** | **Details** |
| --- | --- | --- |
| 1 | Introduction |  |
| 2 | Informed consent |  |
| 3 | Participant information | - Stakeholder group - Participant experience with RWE |
| 4 | Factors influencing the need for RWE in decision-making | - Overarching key question:  “When do you think RWE could be necessary, or desirable, to inform regulatory and HTA decision-making?” - Scheme of a medicine’s lifecycle (incl. potentially varying need for RWE) - Discussion of themes: - Feasibility - Ethical considerations - Generalizability - Epidemiology of disease - Real-world aspects of care - HTA specific considerations - Comparators - Room for other considerations in the need or desire for RWE in decision-making |
| 5 | End | - Consent for re-contact - Room for questions and final remarks participant |
