# Supplementary material for: Multiple Perspectives on the Need for Real‐World Evidence to Inform Regulatory and Health Technology Assessment Decision‐Making: Scoping Review and Stakeholder Interviews
Source: Pharmacoepidemiol Drug Saf. 2025 Jan 7;34(1):e70074. doi: 10.1002/pds.70074 (PMC11706668; doi:10.1002/pds.70074)
Supplement: Supplementary file 4 — Supplementary Material S1. List of factors that increase the desirability or neccessity of RWE in HTA decision‐making. [file PDS-34-e70074-s004.docx]

Multiple perspectives on the need for real-world evidence to inform regulatory and health technology assessment decision-making: scoping review and stakeholder interviews

Supplementary material S1

# **List of factors that increase the desirability or necessity of real-world evidence in health technology assessment decision-making**

Marieke S. Jansen ^1^ ; Olaf M. Dekkers ^1 2 3^ ; Saskia le Cessie ^1 4^ ; Lotty Hooft ^5 6^ ; Helga Gardarsdottir ^7 8 9^ ; Anthonius de Boer ^3 7^ ; Rolf H.H. Groenwold ^1 4^

^1^ Department of Clinical Epidemiology, Leiden University Medical Center, Leiden, the Netherlands
^2^ Department of Endocrinology and Metabolic Disorders, Leiden University Medical Center, Leiden, the Netherlands
^3^ Dutch Medicines Evaluation Board, Utrecht, the Netherlands
^4^ Department of Biomedical Data Sciences, Leiden University Medical Center, Leiden, the Netherlands
^5^ Cochrane Netherlands, University Medical Center Utrecht, Utrecht, the Netherlands
^6^ Julius Center for Health Sciences and Primary Care, University Medical Center Utrecht, Utrecht, the Netherlands
^7^ Division of Pharmacoepidemiology and Clinical Pharmacology, Utrecht Institute for Pharmaceutical Sciences, University Utrecht, Utrecht, the Netherlands
^8^ Department of Clinical Pharmacy, University Medical Center Utrecht, Utrecht, the Netherlands
^9^ Faculty of Pharmaceutical Sciences, University of Iceland, Reykjavik, Iceland

# Table of Contents

1 Questions that can be answered with RWE3

1.1 Epidemiology and care pathways3

1.2 Health technology assessment5

2 Contextual factors that increase the desirability or necessity of RWE11

2.1 Feasibility11

2.2 Ethical considerations14

2.3 Limitations of available evidence16

2.4 Disease & treatment specific aspects21

3 Reference table per individual factor (Table S2)23

4 References25

1. Questions that can answered with RWE and facilitate HTA decision-making
   1. **Epidemiology and care pathways**
      1. Disease & population related aspects
         1. *Incidence, prevalence, event rates*
            Incidence or prevalence of the disease of interest in a certain population. It can also include events associated with the disease of interest, or event rates of infectious diseases.

            *“RWD was included in 28/52 (54%) REAs [relative effectiveness assessments] and was mainly used to estimate melanoma prevalence and/or incidence (28/28 REAs).“* ^82^

            *“Within these submissions, primary care databases have been used to describe the current epidemiology of a disease, to describe current treatment and/or to provide clinical input into CE [cost-effectiveness] models.”* ^75^

            *“In the HAE case, there are two examples where using RWE in the economic model better aligns the cost-effectiveness findings to HAE prophylactic treatment use in clinical practice. First, baseline HAE attack rates observed in the real-world were lower than what was observed in the RCTs. When the cost-effectiveness model was updated with this lower real-world baseline HAE attack rate, the trial-based relative reductions in HAE attacks resulted in fewer attacks averted, thus yielding less favorable cost-effectiveness findings.”* ^61^
         2. *Natural history of a disease*
            The course or progression of a disease over time, from inception to the final outcome (e.g., resolution of the disease or death) under no treatment, or under the current standard of care. Natural history data may also illustrate the burden of disease and unmet needs.

            *“A natural history study is a preplanned observational study with intention to track the course of the disease to identify demographic, genetic, environmental, and other variables that correlate with the disease’s development, progression, and prognosis.” ^113^

            “Patient registries have the potential to illuminate the natural course of disease, provide a source for historical or contemporary controls and assist in the assessment of effectiveness and safety.” ^41^*
         3. *Transition probabilities between disease states*This information relates to the natural history of a disease, but describes the probabilities to transition between disease states specifically. Treatment options may vary depending on disease state, which often shows correspondence with disease severity.

            *“[…] RWD may provide complementary evidence on treatment effects (transcripts b, d, and f), be used to evaluate the health effects over time in the form of utilities, or provide data on transition probabilities between different disease states in pharmacoeconomic models.”* ^80^
         4. *Population characteristics*Distributions of relevant characteristics of the population of interest. Potentially relevant examples include age, sex, disease stages, comorbidities, ethnicity, socioeconomic status, biomarker distributions, etc.

            *“RWD collected from EHRs could also provide information on patient characteristics (e.g., biomarker prevalence) and treatments (e.g., different lines and sequences of therapies, and standard-of-care treatments not selected as comparators in RCTs) in a large patient population.” ^114^*
      2. Treatment related aspects
         1. *Landscape of standard of care and treatment patterns*Description of which, how and when treatments are used in clinical practice for the treatment of the disease of interest. This information may not only illustrate current treatment paradigms (i.e., standard of care), but could also highlight other details that may be specifically relevant to HTA decision-making, such as:

- *Place of the new treatment in standard of care*
- *Thresholds of disease severity at which treatments are prescribed*
- *Management of adverse events*
- *Potential differences in standard of care versus clinical guideline recommendations*
- *Potential differences in standard of care between geographical regions*
- *Off-label use of treatments*

*“RWE can reveal insights into patient quality of life, the actual frequency of disease monitoring in clinical practice, factors that contribute to treatment intolerance or discontinuation, factors that determine treatment choice, patterns of treatment switching and associated outcomes, and health care resource utilization and costs.” ^112^*

- - - 1. *Adherence rates*Information about adherence to the treatment of interest. Adherence rates in routine clinical practice may not only differ significantly from those observed in traditional trials, they can also vary between treatments, and could be an indication of their tolerability.

         *“RWE studies can also provide information about real-world adherence to an intervention and the threshold of disease severity at which point the intervention is prescribed.”* ^69^
      2. *Resource utilization*Description of healthcare use in routine clinical practice for the disease of interest. This information may not only include treatments prescribed, but could also include other types of healthcare use, such as diagnostic testing and the number of visits to outpatient clinics.

         *“Resource use and cost parameters required for modelling should reflect the health system’s service delivery patterns and routine setting, and it is therefore not always appropriate to include resource use from RCTs because the latter are protocol-driven (e.g. regular CT scans or clinical appointments) and not reflective of routine clinical practice. Also, because it is important for NICE to use UK specific costs, sources for resource data are often from NHS-based observational studies, administrative data, chart reviews, listing published by the Department of Health, national data based on healthcare resource groups, such as the Payment by* *Results tariff, the British National Formulary, and sometimes even from expert opinion.” ^51^*
      3. *Cost of care*The costs of healthcare delivered to patients with the disease of interest. This information may include costs of treatments prescribed, diagnostic testing, visits to an outpatient clinic, etc.

         *“In addition to clinical outcomes, RWD databases also enable assessment of health care utilization and associated costs, thus further improving understanding of the broader impact of the use of a particular medication.” ^65^*
  1. **Health technology assessment**
     1. Initial reimbursement: Relative effectiveness assessment (REA) and economic evaluation
        A key aspect of reimbursement decision-making concerns performance of the new treatment relative to treatments already used in standard of care, both in terms of clinical effectiveness and safety. In addition to this relative effectiveness assessment (REA), HTA bodies typically require economic evaluations for guiding decisions related to reimbursement (e.g., “is the new treatment not only safe and effective, but also cost-effective, compared to other available treatments?”). RWE can facilitate the REA and economic evaluation on several levels, and is outlined below.
        1. *Choice of comparators*RWE plays an important role for identifying which comparators the new treatment should be compared to. Relevant comparators are typically based on the standard of care of the country the health technology assessment is performed in, which may not only differ from other countries or geographical regions, but can also change rapidly over time. Clinical guidelines, as well as timely RWE on aspects such as the landscape of standard of care and treatment patterns, will help inform the choice of comparators for the REA and economic evaluation.

           *“Several studies highlighted the usefulness of RWE for HTA purposes. Pietri & Masoura identified 10 guidelines from 73 European HTA agencies or governmental authorities citing RWE. Across these guidelines, there was a consensus that RWE is valuable in providing clinical practice evidence on treatment pathways and comparators […]” ^10^

           “For all interventions, current (inter)national guidelines and standards play an important role in determining which intervention should be chosen as a comparative treatment. Sometimes, multiple interventions could be possible for a specific patient population. Usual care can vary from country to country, or even within one country. Furthermore, usual care may also involve not providing treatment but advising to wait-and-see, either alone or in combination with symptom relief (supportive or palliative care).” ^116^* [translated from Dutch by the authors]

           *“The company also conducted a survey to establish real-world treatment patterns in order to determine relevant comparator treatments used in UK clinical practice”* ^15^
        2. *Transferability assessment*
           RWE can be used to assess whether the results from traditional trials or other studies could be transferable or applicable to the population of interest for the health technology assessment (typically the population of the country that the health technology assessment is performed in). Often, assessments regarding transferability are based on characteristics of patient populations and characteristics of the healthcare system. In some instances, population characteristics data could also be used to reweigh the results of traditional trials, to predict outcomes in populations that health technology assessors are interested in.

           *“Real-world data is already widely used to inform NICE guidance to, for example: […] assess the applicability of clinical trials to patients in the NHS.” ^87^*
           *“The uses of real-world data include: […] predicting outcomes and treatment effects in routine settings, for example, by reweighting results from trials to reflect characteristics of all eligible patients. NICE technology appraisal guidance on pembrolizumab with carboplatin and paclitaxel for untreated metastatic squamous non-small-cell lung cancer used prescribing data from the Cancer Drugs Fund to estimate outcomes weighted by subgroup prevalence.”* ^87^
        3. *Clinical effectiveness*
           Because of their high internal validity, randomized trials are typically the preferred method to assess efficacy and effectiveness of the new treatment compared to other existing treatments in clinical practice. This encompasses both traditional trials and pragmatic trials generating RWE, with the latter potentially generating estimates of effectiveness that better resemble real-world clinical practice, which could be directly relevant to HTA decision-making. However, at the time of initial reimbursement decisions, randomized studies that compare (all) relevant treatments are often not available, or they may not be fully sufficient to inform effectiveness for the health technology assessment for other reasons, such as restricted follow-up times and use of surrogate outcomes.

           *“Several agencies specify that treatment effects used for modelling relative effectiveness should primarily be based on results from RCTs (transcripts b, d, and f). Alternatively, RWD may provide complementary evidence on treatment effects (transcripts b, d, and f)[…]” ^80^

           “All agencies requested RWE for evidence on costs, epidemiological data, and transition probabilities in models. In all of these cases, conclusions on treatment effects based on RWE appeared to play a supplementary role to RCT evidence during the decision-making process.” ^10^*
        4. *Safety*
           Safety outcomes are often rare and may be occurring late after the start of treatment. Traditional pre-approval trials may be too limited in size and duration to detect these outcomes. Additionally, as the decision to treat a patient typically does not involve the potential occurrence of type B side effects, these safety outcomes can be estimated in RWE studies without being plagued by confounding. While evidence from traditional trials may be used to characterize and quantify harms for the health technology assessment, RWE studies (e.g., registries and spontaneous reporting systems) play an important complementary role to inform safety, particularly for rare and late-occurring adverse events.

           *“In contrast, longer study durations and large-scale databases used in RWE studies can reveal rare adverse events (AEs), assess the true incidence and impact of AEs in routine clinical practice, and monitor AEs that emerge after long-term treatment; they can also be a valuable source of information on patient-centered outcomes.”* ^112^

           *“Nonrandomized RWE on drug safety from large healthcare databases and registries is often considered to be more suitable for detecting new safety issues, such as rare serious adverse events that were not seen in registrational clinical trials. Such evidence may be more reliable than that on drug effectiveness, since it is assumed that physicians are typically not considering the risk of unknown or emerging drug adverse events when making prescribing decisions. In that case, patients receiving the medication of interest may have a more similar background risk of the adverse event as patients not receiving the medication, making treatment groups more comparable and less likely to have significant confounding.”* ^47^
        5. *Economic modelling parameters*
           Depending on the HTA body and national regulations, the economic evaluation may encompass a cost-effectiveness analysis, cost-utility analysis, and a budget impact analysis. The economic models that are used for these analyses require input data on various domains, such as incidence and prevalence, clinical effectiveness, safety, quality of life, resource utilization, costs, transition probabilities between disease states, adherence rates, etc. RWE on the topics listed under theme 1.1 can be used as input parameters for these economic models, in addition to evidence from traditional trials.
            *“[…]RWD is directly requested by five HTA agencies for various aspects of PEA [pharmaco-economic analysis][…] agencies recommend that epidemiological data (e.g., incidence and prevalence), direct and indirect costs, and resource use in routine practice be collected from national RWD sources (e.g., claims databases, registries, and hospital databases) (transcripts b, e, and f). Other aspects of the evaluation, such as adherence to treatment and compliance, can also be collected from RWD sources such as registries, databases, ad hoc studies, or epidemiological surveys.” ^80^*
     2. Alternative reimbursement schemes
        1. *Conditional reimbursement schemes*
           Similar to conditional approval in regulatory decision-making, reimbursement may be provided initially on a conditional basis, subject to further evidence being generated to confirm the cost-effectiveness of the treatment of interest. Conditional reimbursement schemes are typically considered for treatments that are highly promising, but present substantial uncertainties, that could be resolved with further evidence collection. RWE studies (e.g., registry studies) can be used to satisfy these post reimbursement conditions.

           *“There has been an increase in the use of expedited access programs by regulatory authorities to facilitate faster patient access to innovative treatments, particularly in oncology and rare diseases, where single-arm trials are common. Following approval through expedited regulatory pathways, HTA bodies are required to make initial reimbursement and/or pricing decisions based on this limited evidence. When substantial uncertainties exist about the clinical evidence for a highly promising treatment, and data collection to resolve the uncertainties is feasible, a conditional reimbursement decision may be given, requiring an outcomes-based managed entry agreement to collect data post reimbursement to resolve the uncertainties, commonly in the form of a nonrandomized study.” ^66^

           “In Europe, RWE is a recognised tool for accelerated access programs across several countries (Gill et al., 2016). One example of a conditional reimbursement scheme that relies on RWE is provided by NICE in the UK. NICE’s observational data unit focuses on commissioning through evaluation (CTE) (this is a type of coverage with evidence development, or CED). CTE enables a number of patients to access treatments that are not widely funded by the UK’s National Health Service (NHS), whilst data on their outcomes (clinical and patient experience) are collected.” ^58^*
        2. *Outcomes-based or pay-for-performance reimbursement schemes*
           In addition to conditional reimbursement schemes or managed entry agreements, other nonconventional schemes for which RWE could be utilized include outcomes-based or pay-for-performance schemes. In these schemes, reimbursement may for example be linked to individual patient outcomes observed in clinical practice, for which RWD is well-suited to track such outcomes.

           *“The final area in which RWE is currently used by payers is in outcomes based contracting, in which payment is linked via rebate levels or some other mechanism to the demonstrated real-world outcomes of patients. Examples from the US include: i) Novartis recently announced an agreement with CMS where CMS will cover Kymriah® (CAR-T therapy) only if patients respond within the first month after treatment; ii) Merck agreed to provide rebate payments to Cigna and Prime Therapeutics for its MS drug Rebif if hospital visits were required due to relapses (QuintilesIMS, 2013).” ^58^*
     3. Other domains and relevant evidence gaps
        1. *Heterogeneity of treatment effects*

The presence of any clinically significant differences in effects among different subpopulations (e.g., those stemming from variations in pharmacogenomics and pharmacokinetics) is not only relevant to patients, but could also guide and optimize reimbursement decision-making for subgroups. However, pre-approval trials are often underpowered to assess heterogeneity of treatment effects. Large datasets obtained from RWE studies could provide a viable alternative.

*“In some areas, RWE had a unique role to play, for example, when assessing safety and efficacy in subgroups of the population, where randomized trials often lack a sufficient number of patients.”* ^13^

*“The uses of real-world data include: […] exploring heterogeneity in intervention effects. NICE technology appraisal guidance on pembrolizumab for treating relapsed or refractory classical Hodgkin lymphoma after stem cell transplant or at least 2 previous therapies used SACT data to model overall survival among those without previous stem-cell transplant.”* ^87^

- - - 1. *Broader impact on the healthcare ecosystem*
         RWE, including qualitative studies, can provide valuable information on the broader impact and effects of introducing a new treatment into the healthcare system. These outcomes extend beyond the direct (health) effects on individual patients, and consider broader implications for the healthcare system, and society as a whole. Several (non-exhaustive) examples of such impacts that may be informed by RWE and could be relevant to consider in HTA decision-making, are:
- Ethical, social or legal impact (e.g., ethical considerations regarding the impact of the treatment on distribution of health care resources, factors preventing treatment access and potential health inequalities, drugs replacing invasive procedures, ethical consequences of criteria for starting preventative medication, impact on care-givers and family)
- Organizational, structural or financial impact (e.g., impact on organization of healthcare service, length of hospital stays, waiting lists, administering treatment at the hospital by trained personnel versus at home, absenteeism from work)
- Population-level effects (e.g., changes in disease prevalence and transmission rates after introduction of a vaccine)
- Occupational and environmental impact (e.g., potential harms for healthcare professionals, waste generation, ecological footprint)

*“Observational real world data can be highly appropriate when there are important uncertainties regarding the real impact on the health care system (organizational, financial and other).” ^40^*

*“Real-world data is already widely used to inform NICE guidance to, for example: […] identify, characterise and address health inequalities” ^87^*

*“In the field of rheumatoid arthritis, for example, patient registries provided data for recent publications on topics as diverse as prescription patterns, disease outcomes, effectiveness and safety of biologicals, health-related quality of life, and socio-economic impacts of the disease.” ^9^

“The following issues might be considered to value the relevance of additional observational data collection. […] Potential harms to health care professionals (for example, radiation) or the environment (dangerous waste) are not adequately covered in available studies.” ^40^*

- - 1. Implementation and monitoring
       1. *Implementation in clinical practice* *and quality of care*
          Post-reimbursement, RWE studies (e.g., registries) can be used to evaluate the implementation, clinical uptake, and appropriateness of utilization of the new treatment in clinical practice (e.g., whether the treatment is prescribed in correspondence with evidence-based guidelines), as well as the impact on the quality of care.

          *“Real-world evidence can be used to: […] provide information on how drugs and health technologies are implemented and managed in the real-world setting” ^20^

          “In line with these concerns, many experts are beginning to consider that collecting observational data after general coverage could be an option to evaluate the appropriateness and quality of care and determine the real impact in the health system, without denying key therapy to patients when uncertainties are not dealing with essential effectiveness or safety issues.” ^40^

          “Many RWD sources, including insurance claims and EHR databases, are used to monitor quality of care.” ^118^

          “Real-world data is already widely used to inform NICE guidance to, for example: […] assess the impact of interventions (including tests) on service delivery and decisions about care.” ^87^*
       2. *Monitoring and re-evaluations*
          Post-reimbursement RWE can serve as valuable complementary evidence to the existing evidence base that was available during the initial assessment. It can be utilized to reassess the relative effectiveness, safety, and cost-effectiveness of a treatment in clinical practice. This becomes particularly pertinent when real-world applicability differs from pre-approval trials, potentially yielding different outcomes. Factors contributing to such differences may include for example variations in treatment protocols between clinical practice and pre-approval trials including concomitant use with other drugs, differences in populations, etc. Additionally, RWE studies enable the capture of long-term outcomes, including unexpected rare or late-occurring adverse events. RWD studies also present opportunities to continuously capture outcomes during the evolving standard of care. HTA decision-makers can leverage these post-reimbursement RWE studies (including those addressing potential evidence gaps listed under ‘other domains and relevant evidence gaps’) to inform updates to reimbursement criteria, or prompt a reconsideration of reimbursement recommendations.

          *“RWE gives HTA/payers the opportunity to reconsider coverage, formulary placement, and price/payment terms in light of how the products are performing in their relevant population.” ^58^

          “Real-world evidence is currently being used in Canada to: […] Inform potential changes in reimbursement criteria for drugs and other health technologies already in use in Canada.” ^20^*

1. Contextual factors that increase the desirability or necessity for RWE in HTA decision-making
   1. **Feasibility**
      1. *Rare populations*
         If the population of interest is extremely rare (e.g., a rare disease or a specific subgroup), it could be impossible to recruit a sufficient number of participants for an adequately powered RCT. An alternative could be to conduct a single arm trial (SAT) and to contextualize the results on the basis of RWE (e.g., a natural history study). Another example includes hybrid RCTs, where RWD are used to augment the control arm of a traditional trial to increase power.

         *“First, a randomized controlled trial for some diseases may not be practical and/or operationally feasible due to scarcity of patients (rare diseases), in which case a single-arm clinical trial may be authorized to compare the investigational product with controls in retrospective or concurrent control cohorts’ data in terms of safety and efficacy evaluation for regulatory decision-making.” ^23^

         “Because it is not always feasible to conduct an RCT, particularly among patients with rare cancers or genomic subtypes, the Food and Drug Administration (FDA) has accepted RWE from expanded-access studies, medical records, and insurance claims.” ^49^

         "However, one of the key issues for drug development in the rare disease space is that there are unique challenges to implementing the gold standard randomized controlled trial design. The small number of patients worldwide with any specific rare disease makes enrolment of a sufficient number of subjects to achieve statistical power in a traditional randomized controlled study difficult if not logistically impossible." ^53^*
      2. *Recruitment difficulties*
         Sometimes, even though the population of interest is not rare per se, there may be recruitment difficulties making a traditional trial difficult to execute. For example, when a treatment is already freely available on the market in the post-approval phase, patients may not want to participate in a traditional trial. In scenarios where recruitment is expected to be extremely challenging and difficult to improve, RWE may be able to provide a more feasible alternative.

         *“Traditional phase IV and other postmarketing studies can be cumbersome and face a myriad of patient enrolment barriers such as changing practice patterns.”* ^84^

         *“[…] providers may not be able to implement randomization because once a product is on the market, patients may be less willing to consent to receiving a randomized treatment. Patients may be resistant to participating in clinical care that diminishes their autonomy and decision-making power.” ^31^*
      3. *Time constraints*
         Traditional trials may take a longer time to conduct in comparison to certain RWE studies. If evidence is needed immediately, use of retrospective RWD (e.g., EHR or claims data) may a more viable alternative. Additionally, patient recruitment is typically faster in prospective observational studies and pragmatic trials than traditional trials.

         *“Integrating RWE into the drug development and approval process has the potential to reduce the time, cost, and patient burden associated with clinical trials while providing clinically relevant information to all stakeholders.” ^43^

         “Appropriate use of RWE for regulatory decision-making holds the promise of generating rapid, less costly evidence by relying on existing clinical practice infrastructure to efficiently identify and follow patients.” ^52^

         “Time can be a barrier for recruiting enough patients to adequately power an interventional study. For example, when measuring long-term or rare outcomes, the time that it would take to recruit a population large enough to conduct a robust study is too long to add relevant, meaningful information to the body of evidence for a product. Additionally, researchers are often concerned about patients dropping out of a study or being lost to follow-up in a long-term study. Use of a control arm can extend the amount of time it takes to recruit a sufficient study population and thus can extend the time it takes to make a drug available for a given use. Any delay in availability of effective treatment options impacts patients with rare or life-threatening diseases with limited treatment options in particular.” ^31^*
      4. *Resource and funding constraints*
         Traditional trials are generally more resource intensive than RWE studies, for example due to strict monitoring schemes, thus requiring a sufficient number of study personnel. If the conduct of a traditional trial is cost-prohibitive (e.g., lack of funding for drugs that are off-patent), RWE studies may provide a more feasible approach.

         *“[…] there are situations where a robust clinical trial is not practicable, for example, because of low recruitment prospects (e.g., for rare diseases), prohibitive anticipated costs and/or resource needs or ethical prohibition (e.g., in cases where there is no established standard of care [SoC] and an RCT would be unethical).” ^91^

         “RWE might be generated more efficiently and with fewer resources, increasing the availability of information that might not otherwise be collected.” ^31^

         “Finally, as retrospective real-world studies use existing data sources, they can be more economical and time efficient than RCTs.” ^89^*
      5. *Long-term outcomes*Traditional trials generally have a limited follow-up time due to prohibitive costs and potential attrition problems associated with conducting trials with long-term follow-up (e.g., multiple years). This could result in the infeasibility to measure long-term outcomes (e.g., mortality) within a trial’s timeframe. In contrast, RWE offers an alternative approach, where patients are typically followed for many years. Alternatively, trialists may opt to utilize surrogate outcomes (see ‘representativeness of endpoint’), where RWE could play a crucial role in confirming the validity of these surrogate outcomes.

         In HTA decision-making, economic modelling is often projected over several decades, much longer than timeframes of traditional trials. Here, RWE can be used as a valuable tool to facilitate extrapolations of trial outcomes beyond trial durations, by informing and validating the assumptions underlying these extrapolations.

         *“Registries may also be particularly useful for tracking effectiveness outcomes for a longer period than those usually realizable with clinical trials. For instance, some registries on growth hormone have tracked children up to the adulthood.” ^90^

         “In addition, patterns of response with I-O therapies are somewhat distinct from other cancer treatments and are often characterized by durable tumor suppression and associated long-term survival. This has led to calls for innovative methodologies and study designs, suggesting that traditional clinical trial approaches may not be optimal for assessing long-term effects of I-O therapy.” ^91^

         “Long-term or late outcomes may not be captured during RCTs, whereas RWE studies can provide a picture of long-term outcomes with CML treatment.” ^112^

         “RWD was included in 22/25 (88%) CEAs [cost-effectiveness assessment] and was primarily used to extrapolate effectiveness of the new drug beyond RCT trial duration to estimate its long-term effectiveness (21/22 CEAs).” ^82^

         “Real-world data has been used in NICE guidance to contextualise clinical trials including for: […] assessing the appropriateness of assumptions about long-term outcomes or treatment effects beyond trial periods: NICE technology appraisal guidance on nintedanib for treating progressive fibrosing interstitial lung diseases used registry data to validate extrapolations of long-term outcomes.” ^87^*
      6. *Rare outcomes*It requires a large study population to be able to detect a rare outcome. It is generally infeasible to include such large populations in traditional trials, as it could severely constrain the time and cost to conduct the trial. Expansive datasets obtained from RWE studies can provide a feasible alternative to study rare outcomes.

         *“They [patient registries] often include larger sample sizes and longer follow-up periods than RCTs, which can allow assessment of rare/long-term outcomes and provide additional safety information after drug approval.” ^89^

         “For low event rate endpoints, RWE can identify rare unanticipated complications better than RCTs, which generally study fewer subjects for shorter time periods.” ^69^

         “EHRs may have the potential to provide clinical investigators and study personnel access to real-time data for review and can facilitate post-trial follow-up on patients to assess long-term safety and effectiveness of medical products. In addition, there are opportunities for long-term follow up of large numbers of patients, which may be of particular importance in studies where the outcome of interest occurs rarely, such as in prophylaxis studies.” ^106^*
      7. *Multiple comparators and treatment combinations*
         It could be relevant to assess the performance of a new treatment against multiple treatment options, combinations, or sequences for the health technology assessment. However, the practicality of conducting numerous RCTs to evaluate all potential options is often constrained by financial, resource, and time limitations. In this context, RWE studies and insights from previously conducted trials offer a pragmatic alternative (e.g., by synthesizing various sources of evidence in a network meta-analysis).

         *“A further consideration is the large number of emerging I-O agents [13]. This rapid expansion may result in a need to compare new agents and new combination regimens against each other and/or against an expanding list of SoC treatments. However, conducting multiple RCTs to facilitate these comparisons may be cost and resource prohibitive.“ ^91^

         “However, randomised controlled trials may not be available for several reasons, including: […] not all treatment combinations (including treatment sequences) can be directly assessed.” ^87^

         “In the context of early phase testing of combination therapies, RWD could be used to form single-agent external control groups against which novel drug combinations could be tested and compared. In this way, RWD-based external control groups would be helpful in assessing the contribution of components in combination regimens. These data would also create the opportunity to augment data collected in pivotal studies of novel drug combinations by potentially replacing single-agent arms in phase III studies, when appropriate.” ^114^*
   2. **Ethical considerations**
      1. *High unmet need*
         In diseases with a high unmet medical need (e.g., a life threatening or severely debilitating disease, without an effective standard of care), it could be unethical to randomize patients to a control arm and deprive them of the new, potentially effective treatment. In these situations, a single arm trial (SAT) may be the most ethical alternative, where RWE could be used to contextualize the results.

         *“For lethal conditions with no available treatments, randomizing patients to receive highly promising treatment may be unethical.” ^48^

         "In addition, the ethics of running a classic placebo controlled trial in a population for whom no standard of care exists is challenging, particularly if a targeted therapy has shown significant potential to decrease disease-related morbidity either pre-clinically or in case reports. For these reasons, many rare disease clinical trials are designed as single arm studies that rely on the use of a control group that is external to the study itself and is based on real-world data." ^53^*
      2. *No equipoise*
         If available evidence shows compelling efficacy of the investigational treatment in comparison to placebo and/or other treatment options, there may no longer be equipoise and it could become unethical to randomize. For example, if evidence from a highly robust phase II trial demonstrates compelling efficacy of the investigational treatment, it may no longer be considered ethical to conduct a traditional phase III randomized trial. Instead, a single arm trial (SAT) contextualized with RWE could be carried out.

         *“For any prospective study design, it is unethical to implement randomization without equipoise between treatments being compared. This means if the intervention of interest is already available on the market and is known to be a better treatment option, then study administrators cannot randomize patients to a less effective treatment.” ^31^

         “There are some clinical circumstances where randomization is impossible to undertake—due to ethical concerns and a state of clinical equipoise may not exist.” ^16^

         “Even in the case of less-rare cancers, recruitment into later-stage I-O studies (i.e., Phase III RCTs) can be challenging as data from earlier studies may be robust enough that investigators feel that clinical equipoise has been lost and are reluctant to enrol into larger trials where SoC has already been surpassed.”* ^91^
      3. *Vulnerable populations*
         In certain vulnerable populations, such as children and incapacitated patients, recruitment of participants in trials can be viewed as unethical as these populations may not be able to come to a well-informed decision to participate by themselves. Furthermore, participation of pregnant women in trials can be considered unethical due to potential teratogenic effects on the foetus. These populations are often omitted from pre-approval trials (see also ‘representativeness of patient characteristics’). RWE may be able to provide evidence in populations where trials may be more ethically difficult to conduct.

         *“Moreover, conducting clinical trials is not always feasible or ethical for certain diseases/disorders (such as rare diseases) or patient populations (such as children, pregnant women, seniors).” ^55^

         “RWE has also been particularly effective when the outcome of interest is rare, in cases where a very long follow-up period is required to assess the health outcomes, or when it is difficult to perform randomized controlled trials (RCTs), such as in pediatric or pregnant populations.” ^7^

         “Furthermore, more than half of the patients with rare diseases are children, but there are often ethical issues when enrolling children in clinical trials. Accordingly, there is a continued need for exploring and using RWD and RWE to help demonstrate the safety and efficacy of orphan drugs.” ^113^*
      4. *Other ethical considerations*
         There may be other ethical considerations limiting the conduct of an RCT. For example, patients or their care providers may be unwilling to participate in a randomized study if a certain treatment is perceived as the most optimal one (even if scientifically unproven).

         *"The official Health Canada notification lists 3 scenarios for which RWE submissions are encouraged: […] for drugs or diseases where clinical trials are considered unethical (eg, to extrapolate dosages from animal studies to treat humans exposed to chemical or biological threats during emergencies)." ^4^

         “Randomised trials may not be available for several reasons, including: […] patients are unwilling to be allocated to one of the interventions in the trial; healthcare professionals are unwilling to randomise patients to an intervention which they consider less effective.” ^87^*
   3. **Limitations of available evidence**
      1. *Generalizability*
         Although the traditional clinical trial is considered the gold standard to evaluate benefit-risk because of their ability to reduce confounding though randomization, they are often conducted under tightly controlled conditions in populations that may not be representative of everyday clinical practice. This can limit generalizability of results, and is often referred to as the main reason why sometimes efficacy estimates do not translate well into clinical practice (also described as the “efficacy-effectiveness gap”). RWE has the potential to complement traditional trial evidence by providing more generalizable evidence. Below, several aspects of traditional trials are outlined that may lead to reduced generalizability but could be overcome by RWE.

         *“[…] properly performed analyses of real-world data can provide valuable complementary data, which can be used to address the ‘generalizability’ limitations of RCTs and provide evidence on the external validity of their findings. In the case of EGFR mutation positive NSCLC, real-world studies have confirmed that the efficacy and safety profiles of EGFR TKIs seen in RCTs are applicable to real-world populations, and provided valuable evidence on the activity of EGFR TKIs in patient populations not routinely included in RCTs, including elderly patients, patients with brain metastases and, for afatinib, patients with uncommon mutations.” ^89^

         “RWE offers less constrained study designs and broader populations as compared to more traditional RCTs. These attributes offer the possibility of obtaining evidence that more accurately reflects the “true” outcomes of care in real-world care settings, where patients, clinicians, treatment regimens, and all facets of care may be more representative than those involved in the highly controlled RCTs and other trials traditionally used for drug development studies.” ^58^

         “One of the ways in which RWD/RWE can be used to demonstrate drug effectiveness is the pragmatic clinical trial. This type of clinical trial is close to clinical practice in trial designs including less stringent eligibility criteria and endpoints and visit-schedules which can be embraced in routine clinical practice for a targeted disease, allowing for the generalization of research findings to patients in the real-world.” ^68^*
         1. *Representativeness of endpoint*
            Traditional trials sometimes include surrogate or intermediate endpoints which may not necessarily correlate well with clinically relevant endpoints, potentially reducing generalizability of results. RWE studies can be used to validate surrogate endpoints and confirm if the treatment also has the desired effect on clinical outcomes relevant to patients. However, RWE can also be used to identify candidate surrogate outcomes if clinical outcomes are not feasible to include in the setting of an RCT (e.g., the clinical outcome of interest takes too long to follow up).

            *“The endpoints used in RCTs may not be directly relevant to patients and clinicians, especially surrogate endpoints used as substitutes for clinical outcomes that take a long time to develop. For example, 36 of 54 new oncology treatments approved from 2008–2012 used surrogate endpoints for approval, but roughly four years after approval, only 14% showed improvement in overall survival (OS). Half (50%) of those newly approved drugs revealed no survival benefit, with no data available for the remainder.” ^28^

            "RWE may provide supportive data that has greater external validity, as well as providing information on subpopulations, off-label use, misuse, adherence, and to validate surrogate outcomes." ^19^*
         2. *Representativeness of patient characteristics*
            Traditional trials often include homogeneous populations that may not be representative of all patients who could benefit from the treatment in daily practice. They may have strict eligibility criteria, but also are typically conducted at academic/teaching hospitals, which may only treat a selection of patients. This could result in relevant differences between trial populations and real-world patient populations in terms of age, sex, disease severity, patient history and comorbidities, ethnicities, socioeconomic status, etc. In HTA decision-making, this problem could be especially pertinent, as these populations should not only resemble real-world clinical practice populations, but should specifically be similar to those for whom they provide coverage for (e.g., the country that the health technology assessment is performed in). RWE studies can be utilized to evaluate effects of a treatment, including quality of life outcomes, in patient populations that are more representative of those in clinical practice, and specifically of those in the country that the HTA is performed in, or in specific subgroups not studied or underrepresented in pre-approval trials (e.g., children, elderly, patients with worse disease severity, patients with multiple comorbidities).

            *“Real-world studies reflect clinical experience across a broader and more diverse distribution of patients than prospective RCTs and, unlike RCTs, can provide insight into real-world treatment patterns, including dosing, compliance, adherence, off-label use and the balance between efficacy and safety in patient groups not included in RCTs.” ^89^

            "In the United States, RCTs involving patients with cancer represent less than 5% of U.S. adults with cancer. Patients in RCTs are also younger, healthier, and less diverse than the other 95% of patients with cancer." ^49^

            “For example, RCTs used in the regulatory process often have narrow eligibility criteria and take place in highly selected care settings; such RCTs may not reflect the patient populations or processes of routine clinical practice. Furthermore, even among patients who meet trial eligibility criteria, the patients enrolled in regulatory trials and those seen in routine clinical practice may have a different distribution of effect modifiers (characteristics over which treatment effectiveness varies); in such cases, the ”true” treatment effect in the (usually ill-defined) population underlying a regulatory trial can be different from the “true” treatment effects in the “real-world” populations where the interventions compared in the trial will be considered for use. Furthermore, the narrow eligibility criteria of regulatory trials, in combination with highly selected care settings, may mask elements of risk in safety events that are recognized only after product has become widely used in diverse patient populations and settings. These limitations of RCTs motivate the interest in complementing RCT evidence with RWD analyses […].” ^24^

            “Background event rates or natural history data from trials may sometimes over or underestimate event rates in the target population because of selective recruitment. In some cases, there may be value in performing studies using routinely collected data rather than relying on published evidence that has lower applicability to the research question.” ^87^*
         3. *Representativeness of patient behaviour*
            Adherence or compliance in traditional trials is often supported by intensive efforts and may differ from adherence by patients in every day clinical practice. Furthermore, patients behave differently when they know they are being observed (i.e., “Hawthorne effect” or “observer effect”). Patients who participate in traditional trials may also differ in their willingness to seek treatment in comparison to other patients in clinical practice. RWE is more likely to reflect patient behaviours, including adherence rates, found in clinical practice (e.g., RWD from healthcare databases typically involve patients and healthcare providers unaware of being studied).

            *“RWE is useful for comparing the effectiveness of various interventions in the real world, where patients are not being closely followed or encouraged to adhere to treatment like they are in clinical trials […].” ^69^

            “A key advantage of healthcare databases is that they record the routine operation of a system. Patients and providers are not aware that they might be studied using these data.“ ^99^

            “However, the internal validity attained in these trials is often achieved at the expense of uncertainty about generalizability […] adherence to therapies may be supported by intensive efforts that are infeasible in practice.” ^100^

            “Clinical trial protocols may result in different behavior than typical clinical practice.” ^7^

            “For example, patients enrolled in RCTs are typically more homogeneous than patients in the real-world and are more likely to be adherent to therapy.” ^61^*
         4. *Representativeness of treatment setting*
            The setting of traditional trials may not be representative of the diverse clinical practice settings and conditions where the treatment may be delivered in the real world (e.g., highly selected care or research settings vs the general practitioners office, differences in professionals delivering care, practice setting differences between countries, etc.). Analysis of RWD can provide evidence on treatment outcomes in settings that are more applicable to real world clinical practice than evidence from traditional trials.

            *“RWE has the potential to provide useful information regarding the use and/or outcomes of a given treatment within a setting most relevant to UK routine practice […]” ^15^

            “[…] RWD provides an opportunity to enrol more diverse patient populations from a range of treatment settings.” ^76^

            “RW data may provide clear advantage for understanding outcomes of treatment, for example, for patients excluded from trials, patients in actual clinical practice settings (vs. research settings), and patients whose treatment is not determined by trial protocol or practice guidelines.” ^50^*
         5. *Representativeness of treatment protocol*
            Treatment protocols used in traditional trials can be restrictive and may not resemble how treatment is applied in clinical practice. For example, there may be differences in dosing regimen, use of co-medication, monitoring, visits to the clinic, use of diagnostic testing and overall clinical support. RWE better reflects care delivered in routine clinical practice.

            *“Compared with clinical trial data, RWE may be considered a better representation of local patient demographics, more reflective of clinical practice, and potentially be closer aligned with contemporary treatment patterns.” ^15^

            “By design, patients in RCTs are highly selected and cared for, with active encouragement to adhere to assigned treatments, close monitoring for disease progression, and optimal testing.” ^28^

            “Adherence, procedures, testing, patient eligibility criteria, “placebo effects”, structured treatment regimens and scheduled clinic visits in RCTs are highly regimented and inherently different from routine clinical practice.” ^52^*
      2. *Less robust trial evidence*
         If the robustness of the evidence from pre-approval trials is limited (e.g., if it was based on a very small sample size or short follow-up, or if there are concerns in terms of methodological quality), RWE could support decision-making, especially if improving on previous trial designs or conducting new trials is difficult.

         *"Post-authorisation studies that are performed in patient registries wherein patients are recruited based on a disease (i.e., disease registry) rather than based on a specific drug exposure can be a useful tool to address uncertainties at the time of marketing authorisation (MA). These disease registries may prove of particular relevance in the case of orphan serious/life-threatening diseases such as cystic fibrosis (CF) where the clinical trials supporting MA could be of limited size and duration of treatment. Also, in certain populations, efficacy data may have some residual uncertainties stemming from the limited populations and feasibility reasons at the time of initial approval." ^37^

         "Products intended for rare diseases are often studied in uncontrolled trials and the size of the safety and efficacy datasets at time of marketing authorisation application is small. In these cases, follow-up for efficacy and safety may be needed, and PAES and PASS are often imposed for post-authorisation evidence generation. These are frequently and preferentially performed on the basis of existing patient registries." ^39^

         “Even if randomised evidence is available, it may not be sufficient for decision making in the NHS for several reasons including: […] trials were of poor quality.” ^87^*
      3. *Cross-over issues*
         If, in an RCT, a large number of participants cross over from one treatment arm to another, a potential treatment effect may become diluted and subsequently difficult to detect in an intention-to-treat analysis, yet the per-protocol effect may be biased. Large cross-over effects can thus pose a problem in answering efficacy and effectiveness questions with RCTs. Some suggest RWE could provide an alternative if cross-over effects are expected to be large in traditional trials.

         *“Furthermore, RWD may be important in settings where patients in RCT control groups frequently switch across to the investigational treatment arm. In some cases, patients are switched inadvertently, while in other cases patients are allowed to cross over from the control group to the investigational treatment arm because of ethical issues, such as in the study of panitumumab in metastatic colorectal cancer.” ^114^

         “Even if trials are available, they may not be directly applicable to the research questions or routine care in the NHS because of: […] methods used to address post-randomisation events such as treatment switching, loss to follow up or missing data.” ^87^

         “[…] patients in RCTs may discontinue, augment, or switch products or fail to appear for clinic visits, introducing post-randomization confounding.” ^52^*
      4. *Limited existing knowledge*
         Although epidemiological data to provide clinical context is generally considered helpful for decision-making in any disease area, this could potentially play an even larger role in those diseases for which there is still very little known about the natural history of disease (e.g., in certain orphan diseases).

         *"Due to the low disease prevalence, high disease severity, small and heterogeneous patient populations and limited knowledge of the disease natural history, the overall clinical evidence submitted at the time of marketing authorisation application for these medicines is often limited. […] Patient registries have been recognised as potentially valuable sources of data to address these challenges and support regulatory decision-making on medicines, independent from the original purpose for which they have been established." ^64^*
      5. *Absence of head-to-head trials*While randomized studies are generally the preferred method for assessing the relative effectiveness of a new treatment against the standard of care (as outlined in theme 1), head-to-head trials with an active comparator arm may be unavailable. Instead, pre-approval trials often compare the investigational treatment with a placebo arm. In such cases, RWE studies as well as evidence from prior trials can facilitate indirect comparisons between the new treatment and standard of care (e.g., in a network meta-analysis).

         *“While nearly 50% of treatments come to the marketplace with placebo as the active comparison, payers do not reimburse for placebo or see it as their comparator of interest. They are making decisions about the new treatment as compared with the existing marketed and dominant method(s) of treatment.” ^34^

         “No randomized head-to-head trials comparing second-line nilotinib with dasatinib have been conducted; thus, RWE studies such as this one are an important avenue for obtaining comparative analyses.” ^112^

         “The uses of real-world data include: […] incorporating into evidence synthesis, for example, informing priors, increasing power or filling evidence gaps in a network meta-analysis (NICE Decision Support Unit 2020, Sarri et al. 2020).”* ^87^
      6. *Active comparator not relevant*
         Even when head-to-head trials are available, the chosen comparators may not be relevant for the health technology assessment. For instance, the standard-of-care arm in the trial might not align with the standard of care in the country where the assessment is conducted, as standards can vary between countries. In addition, in rapidly evolving disease areas, the selected comparator in the pre-approval trial may become outdated before the trial concludes. In such scenarios, RWE and evidence from previous trials hold potential to inform relative effectiveness (e.g., in a network meta-analysis, or via the use of external comparator arms).

         *“Once medical technologies are used routinely or in pilot projects, the opportunities for real-world data are greater and include: […] providing head-to-head comparisons with preferred comparators. NICE technology appraisal guidance on mogamulizumab for previously treated mycosis fungoides and Sezary syndrome used HES data to provide a UK-specific standard-of-care comparator arm to the intervention arm of a randomised controlled trial.” ^87^

         “Further, RCTs can be slow and costly to conduct and analyze; consequently, the comparator standard-of-care arm chosen when the trial is designed may not reflect the standard-of-care used when the trial is completed.” ^89^

         “This does not reflect the complexity of care in the real world, including the reality of differing and evolving treatment sequences and modalities. RCTs often compare a new drug with the standard of care. However, in oncology, the standard of care for a given malignancy or subpopulation can change rapidly. Moreover, standard of care interventions can differ across countries, regions, or centers, often based on reimbursement and access issues.” ^4^*
      7. *Relevant outcomes not included in available trials*
         In HTA decision-making, the value of a treatment is often determined on the basis of patient relevant outcomes and quality of life measures (utilities). If the outcomes considered in pre-approval trials fail to encompass relevant parameters necessary for the REA and economic evaluation, RWE can serve as a valuable source of complementary evidence to fill these gaps.
          *“In contrast, longer study durations and large-scale databases used in RWE studies can reveal rare adverse events (AEs), assess the true incidence and impact of AEs in routine clinical practice, and monitor AEs that emerge after long-term treatment; they can also be a valuable source of information on patient-centered outcomes.” ^112^*

         *“RWD was included in 22/25 (88%) CEAs and was primarily used to extrapolate effectiveness of the new drug beyond RCT trial duration to estimate its long-term effectiveness (21/22 CEAs). Additionally, RWD was included to estimate costs associated with drugs (12/22), estimate resource use (8/22) and determine utilities using quality-of-life information (4/22).” ^82^*
   4. **Disease & treatment specific aspects**
      1. *Complex treatment settings*For complex and innovative treatments, the collection of RWE throughout the medicine’s lifecycle may be of extra importance. Advanced therapy medicinal products (ATMPs) are often “first-in-class” products for which the biological mechanism is not yet well characterised and long-term effects are unknown. An example includes gene therapies, where long-term follow-up by utilizing a RWD could be valuable.

         *“In certain instances, for example, with new treatments such as gene therapies, regulatory agencies institute long-term requirements (i.e. 5-, 10-, or 15-year) to understand the durability and safety profile of the treatment. In such instances it is useful to explore new methods of collecting data without requiring patients to return to the clinical site over an extended period of time.” ^1^

         “The conduct of post-authorisation studies is particularly important in the case of advanced therapy medicinal products (ATMPs) as these are often “first-in-class” products, for which at time of marketing authorisation the biological mechanism is not yet fully characterised, and the long-term safety is unknown. A PAES should provide confirmatory data based on use of these medicinal products in real-world clinical settings.” ^64^

         “Continuous and strategic RWD collection may be useful to regulators seeking to monitor the long-term safety of early-administered, onetime therapies like Zolgensma […]” ^32^*
      2. *Vaccine research*
         In the case of vaccines, there can be challenges related to evaluating benefit-risk on the basis of RCT evidence, specifically regarding non-serological outcomes. For example, certain clinical outcomes may take a very long time to develop, herd effects are hard to capture in RCTs, and variability in disease transmission between areas and populations may complicate the interpretation of vaccine efficacy. RWE may be able to provide valuable complementary evidence to help fill certain evidence gaps.

         *“For example, in the special case of vaccines, RWE is particularly important to evaluate herd effects, generally not captured in RCTs.” ^10^

         “Nonrandomized RWE from a large integrated healthcare system was also used to support the long-term effectiveness of a vaccine in preventing herpes zoster and related complications.” ^47^

         “For example, CBER has used CMS data to evaluate the comparative effectiveness of preventative vaccines, including standard-dose and high-dose; egg-based, cellbased, and adjuvanted influenza vaccines; and the effectiveness of a herpes zoster vaccine.” ^105^

         “Regulatory authorities have accepted RWE in marketing authorization decisions in […] effectiveness and dose finding of vaccines, e.g. rabies.” ^11^*
      3. *Changing drug effectiveness over time*
         For some treatments, the effectiveness of the intervention may change over time as users become more experienced (i.e., learning effects). This could also play a role for certain ATMPs, like Chimeric antigen receptor (CAR) T-cell therapy, where a patient’s own immune cells are modified to target cancer cells. As experience accumulates and processing of cells is optimized increasingly over time, efficacy information from pre-approval trials may become less relevant. RWE could provide a practical approach to investigate the effectiveness of these interventions over time.

         *“We have discussed above how chimeric antigen receptor T effectiveness may depend on the production process. As experience accumulates, and cell processing is increasingly optimized over time, the biological effects of these cell products will shift and efficacy information from past clinical trials becomes less relevant.” ^33^

         “Even if trials are available, they may not be directly applicable to the research questions or routine care in the NHS because of: […]; learning effects (that is, the effect of an intervention changes over time as users become more experienced).” ^87^*
2. Reference table per individual factor

**Table S2 References per individual factor**

| **Themes, subthemes and individual factors** | | | **References** | **Counts ^a^ (%)** |
| --- | --- | --- | --- | --- |
| **Theme: Questions that can be answered with RWE** | | | |  |
| **Subtheme 1: Epidemiology and care pathways** | | |  |  |
|  | Disease & population related aspects | |  |  |
|  |  | Incidence, prevalence, event rates | ^1, 10, 12, 15, 19, 21, 34, 39, 52, 58, 61, 64, 75, 80, 82, 84, 87, 94, 95, 98^ | 20 (17%) |
|  |  | Natural history of a disease | ^1, 3, 5, 8, 10, 12, 13, 15, 16, 19-21, 26, 27, 33-36, 39, 41-45, 47, 52-54, 57, 58, 62, 64, 67, 72, 75, 77, 78, 80, 82, 84-88, 90-92, 94, 96, 98, 107, 109, 110, 113, 114, 116, 118^ | 57 (48%) |
|  |  | Transition probabilities between disease states | ^80, 87^ | 2 (2%) |
|  |  | Population characteristics | ^1, 2, 5, 7, 8, 15, 22, 25, 35, 42, 47, 57, 60, 61, 63, 67, 68, 70, 79, 87-91, 94, 95, 97, 101, 106, 109, 111, 112, 118^ | 33 (28%) |
|  | Treatment related aspects | |  |  |
|  |  | Landscape of standard of care & treatment patterns | ^1, 2, 5, 10, 12, 13, 15, 16, 21, 26, 35, 36, 39, 40, 42, 44, 57, 61, 64, 65, 67, 75, 79, 82, 84-87, 89, 94, 98, 101, 112, 114^ | 34 (29%) |
|  |  | Adherence rates | ^19, 25, 36, 40, 47, 50, 54, 57, 58, 67, 69, 80, 81, 87, 89, 96, 103, 112, 114^ | 19 (16%) |
|  |  | Resource utilization | ^1, 9, 10, 12, 13, 15, 19, 20, 24, 26, 27, 29, 31, 32, 36-40, 42, 44, 49-52, 54, 56, 57, 60, 61, 64, 65, 67, 69, 71, 75, 77, 79-83, 85-87, 89, 91, 94, 96-99, 112, 116, 118^ | 55 (47%) |
|  |  | Cost of care | ^6, 10-13, 15, 16, 19, 21, 22, 24, 25, 34, 42, 50, 51, 54, 56-61, 65, 67, 69, 72, 73, 75, 80, 82, 83, 87, 94, 96, 99, 101, 104, 112, 114, 116, 118^ | 42 (36%) |
| **Subtheme 2: Health technology assessment** | | |  |  |
|  | Initial reimbursement: REA and economic evaluation | |  |  |
|  |  | Choice of comparators | ^10, 15, 19, 116^ | 4 (3%) |
|  |  | Transferability assessment | ^87^ | 1 (1%) |
|  |  | Clinical effectiveness | ^6, 7, 9-11, 13-15, 17, 20, 25-29, 32, 33, 36-40, 42, 44, 47-50, 54, 55, 57, 58, 60-62, 64, 65, 67, 69, 71, 72, 80-82, 84-91, 94, 95, 97-102, 104, 105, 108, 110-112, 115, 116^ | 68 (58%) |
|  |  | Safety | ^1, 4-7, 9, 11-22, 24-26, 29-35, 37-49, 52, 54-65, 67, 69, 71-74, 76-79, 81, 83-89, 91, 92, 95-97, 99-105, 110-115, 117, 118^ | 92 (78%) |
|  |  | Economic modelling parameters | ^10, 13, 15, 19, 40, 42, 51, 57-59, 61, 67, 73, 80, 82, 87, 94, 116^ | 18 (15%) |
|  | Alternative reimbursement schemes | |  |  |
|  |  | Conditional reimbursement schemes | ^40, 42, 57, 58, 60, 66, 80, 82, 101, 104^ | 10 (8%) |
|  |  | Outcomes-based or pay-for-performance schemes | ^7, 12, 22, 36, 38, 54, 58, 69, 90, 92, 94, 104^ | 12 (10%) |
|  | Implementation and monitoring | |  |  |
|  |  | Implementation in clinical practice and quality of care | ^7, 20, 39, 40, 57, 64, 65, 83, 87, 97, 100, 103, 114, 118^ | 14 (12%) |
|  |  | Monitoring and re-evaluations | ^12, 20, 22, 30, 31, 40, 42, 50, 57, 58, 60, 64, 82, 86, 87, 104, 112^ | 17 (14%) |
|  | Other domains and relevant evidence gaps | |  |  |
|  |  | Heterogeneity of treatment effects | ^7, 11-13, 18, 19, 25, 27, 35, 40, 54, 57, 58, 63, 64, 67, 70, 71, 77, 79, 83, 85-87, 89, 90, 92, 94, 96, 98, 103, 115, 117, 118^ | 34 (29%) |
|  |  | Broader impact on the healthcare ecosystem | ^2, 9, 40, 42, 57, 58, 61, 69, 83, 87, 97^ | 11 (9%) |

*Table continues on the next page*

| **Theme: Contextual factors that increase the desirability or necessity of RWE in HTA decision-making** | | | | |
| --- | --- | --- | --- | --- |
| **Subtheme 1: Feasibility** | | |  |  |
|  | Rare populations | | ^1, 3-8, 10-12, 15, 16, 18-21, 23, 26, 27, 29-31, 33, 35-49, 52, 53, 55-57, 63, 64, 66, 68, 72-74, 78-80, 84, 86-88, 91-93, 95-98, 100, 102-105, 111, 113-116, 118^ | 75 (64%) |
|  | Recruitment difficulties | | ^4, 7, 49, 64, 68-70, 84, 94, 103, 115^ | 11 (9%) |
|  | Time constraints | | ^3, 6, 7, 16, 25, 26, 29-31, 33, 35, 43, 47, 48, 52, 55, 61-63, 65, 67, 69-72, 76, 79, 83, 86-91, 95, 97, 99, 100, 102-104, 110-112, 114, 115^ | 46 (39%) |
|  | Resource constraints | | ^6, 7, 16, 22, 25-27, 29-31, 33, 35, 43, 48, 50, 52, 61, 63-65, 67, 69-72, 76, 79, 83, 87, 89, 91, 92, 98, 100, 102, 103, 110-112, 114, 115^ | 41 (35%) |
|  | Long-term outcomes | | ^1, 4-7, 10, 15, 16, 19-21, 25, 27, 29-33, 36-40, 42-45, 47, 50, 51, 54, 57-59, 61, 63-67, 69, 70, 72, 78-83, 86, 87, 89-91, 94, 96-98, 101-103, 106, 110-112, 114, 116, 118^ | 68 (58%) |
|  | Rare outcomes | | ^3, 4, 6, 7, 14, 16, 19, 25, 26, 29-31, 33, 35, 40, 43, 47, 48, 50, 52, 55, 59, 61-63, 65, 67, 69-72, 76, 79, 81, 83, 84, 86-91, 95, 97, 99, 100, 102-104, 106, 110-112, 114, 115, 117, 118^ | 57 (48%) |
|  | Multiple comparators and treatment combinations | | ^4, 8, 26, 50, 57, 68, 87, 91, 114^ | 9 (8%) |
| **Subtheme 2: Ethical considerations** | | |  |  |
|  | High unmet need | | ^1, 3, 5, 6, 8, 10, 12, 16-19, 21, 23, 26, 27, 29, 31, 35, 37, 40, 41, 43, 44, 46-48, 50, 51, 53, 57, 58, 64, 65, 68, 72, 73, 78, 80, 84, 86-88, 91-96, 99, 102, 109-111, 113-115^ | 56 (47%) |
|  | No equipoise | | ^16, 19, 27, 31, 87, 91^ | 6 (5%) |
|  | Vulnerable populations | | ^7-9, 14, 19, 30, 37-39, 46, 55, 57, 64, 68, 77, 78, 85, 87, 96, 104, 110, 113^ | 22 (19%) |
|  | Other ethical considerations | | ^4-6, 18-20, 29, 31, 33, 40, 55, 57, 66, 78, 101, 105^ | 16 (14%) |
| **Subtheme 3: Limitations of available evidence** | | |  |  |
|  | Generalizability | | ^6, 7, 14, 19, 24, 33, 41, 45, 49, 50, 57, 58, 63, 98, 99, 103^ | 16 (14%) |
|  |  | Representativeness of endpoint | ^1, 6, 7, 19, 21, 26-31, 44, 46, 47, 60-62, 66, 68, 77, 79-81, 87, 88, 99, 102, 103, 108^ | 29 (25%) |
|  |  | Representativeness of patient characteristics | ^1, 4, 6, 7, 10, 12, 14-16, 19, 20, 22, 24-32, 34, 35, 37, 39, 40, 43, 47-50, 52, 54, 55, 57, 58, 61-72, 76-84, 86, 87, 89-91, 93-96, 98-104, 110-112, 114, 115, 117, 118^ | 80 (68%) |
|  |  | Representativeness of patient behaviour | ^7, 19, 24, 25, 27, 28, 30, 31, 47, 52, 61, 69, 77, 87, 89, 90, 99, 100, 112^ | 19 (16%) |
|  |  | Representativeness of treatment setting | ^6, 7, 15, 17, 20, 24, 27-31, 35, 47, 50, 55, 58, 63, 76, 84, 87, 90, 100, 102, 117^ | 24 (20%) |
|  |  | Representativeness of treatment protocol | ^4, 15, 19, 24-28, 30, 31, 48, 50, 52, 57, 58, 63, 65, 66, 68, 77, 80-82, 84, 87, 90, 99, 100, 102, 111^ | 30 (25%) |
|  | Less robust trial evidence | | ^51, 87, 115^ | 3 (3%) |
|  | Crossover issues | | ^16, 51, 52, 87, 114^ | 5 (4%) |
|  | Limited existing knowledge | | ^64^ | 1 (1%) |
|  | Absence of head-to-head trials | | ^1, 10-13, 15, 17, 19, 22, 25, 29, 32, 34, 38, 40, 41, 50, 56-58, 60, 61, 65, 67, 69, 71, 73, 75, 77, 79-83, 89, 94, 96, 98, 101, 105, 112, 114, 116^ | 43 (36%) |
|  | Active comparator not relevant | | ^4, 19, 40, 47, 51, 57, 61, 66, 69, 81, 87, 89, 114^ | 13 (11%) |
|  | Relevant outcomes not included in available trials | | ^1, 4, 7, 10, 12, 15, 16, 20, 26, 30-40, 42, 45, 50, 51, 54, 57-59, 62, 65, 78-83, 87, 92, 95, 96, 98, 100-102, 112^ | 45 (38%) |
| **Subtheme 4: Disease & treatment specific attributes** | | |  |  |
|  | Complex treatment settings | | ^19, 21, 32, 38, 39, 44, 45, 57, 64, 87, 88^ | 11 (9%) |
|  | Vaccine research | | ^10, 11, 36, 45, 47, 95, 96, 105^ | 8 (7%) |
|  | Changing drug effectiveness over time | | ^33, 40, 87^ | 3 (3%) |

*This table outlines the factors and themes identified in the scoping review, and the references that each factor was based upon.
^a^ Counts of the references that contributed to each factor, and between brackets % of the total 118 references.*

1. References

1. Health Canada & Canadian Society for Pharmaceutical Sciences - Use of Real World Data/Evidence to Inform Regulatory Decision Making. *J. Pharm. Pharm. Sci.* **23**, 1s-47s (2020).

2. Annemans, L. & Makady, A. TRUST4RD: tool for reducing uncertainties in the evidence generation for specialised treatments for rare diseases. *Orphanet J. Rare Dis.* **15**, 127 (2020).

3. Arondekar, B. *et al.* Real-World Evidence in Support of Oncology Product Registration: A Systematic Review of New Drug Application and Biologics License Application Approvals from 2015-2020. *Clin. Cancer Res.* **28**, 27-35 (2022).

4. Azoulay, L. Rationale, Strengths, and Limitations of Real-World Evidence in Oncology: A Canadian Review and Perspective. *Oncologist* **27**, e731-e738 (2022).

5. Bakker, E., Plueschke, K., Jonker, C.J., Kurz, X., Starokozhko, V. & Mol, P.G.M. Contribution of Real-World Evidence in European Medicines Agency's Regulatory Decision Making. *Clin. Pharmacol. Ther.* **113**, 135-151 (2023).

6. Baumfeld Andre, E., Reynolds, R., Caubel, P., Azoulay, L. & Dreyer, N.A. Trial designs using real-world data: The changing landscape of the regulatory approval process. *Pharmacoepidemiol. Drug Saf.* **29**, 1201-1212 (2020).

7. Beaulieu-Jones, B.K. *et al.* Examining the Use of Real-World Evidence in the Regulatory Process. *Clin. Pharmacol. Ther.* **107**, 843-852 (2020).

8. Bolislis, W.R., Fay, M. & Kuhler, T.C. Use of Real-world Data for New Drug Applications and Line Extensions. *Clin. Ther.* **42**, 926-938 (2020).

9. Bouvy, J.C., Blake, K., Slattery, J., De Bruin, M.L., Arlett, P. & Kurz, X. Registries in European post-marketing surveillance: a retrospective analysis of centrally approved products, 2005-2013. *Pharmacoepidemiol. Drug Saf.* **26**, 1442-1450 (2017).

10. Bowrin, K., Briere, J.B., Levy, P., Millier, A., Clay, E. & Toumi, M. Cost-effectiveness analyses using real-world data: an overview of the literature. *J. Med. Econ.* **22**, 545-553 (2019).

11. Breckenridge, A.M., Breckenridge, R.A. & Peck, C.C. Report on the current status of the use of real-world data (RWD) and real-world evidence (RWE) in drug development and regulation. *Br. J. Clin. Pharmacol.* **85**, 1874-1877 (2019).

12. Brixner, D. *et al.* Payer perceptions of the use of real-world evidence in oncology-based decision making. *J. Manag. Care. Spec. Pharm.* **27**, 1096-1105 (2021).

13. Brown, J.P., Douglas, I.J., Hanif, S., Thwaites, R.M.A. & Bate, A. Measuring the Effectiveness of Real-World Evidence to Ensure Appropriate Impact. *Value Health* **24**, 1241-1244 (2021).

14. Brown, J.P., Wing, K., Evans, S.J., Bhaskaran, K., Smeeth, L. & Douglas, I.J. Use of real-world evidence in postmarketing medicines regulation in the European Union: a systematic assessment of European Medicines Agency referrals 2013-2017. *BMJ Open* **9**, e028133 (2019).

15. Bullement, A. *et al.* Real-world evidence use in assessments of cancer drugs by NICE. *Int. J. Technol. Assess. Health Care*, 1-7 (2020).

16. Burcu, M. *et al.* Real-world evidence to support regulatory decision-making for medicines: Considerations for external control arms. *Pharmacoepidemiol. Drug Saf.* **29**, 1228-1235 (2020).

17. Burns, L. *et al.* Real World-Evidence for Regulatory Use Decision Aid: An Interactive Tool To Inform Clinical Development and Regulatory Strategies. *Adv. Ther.* **39**, 4772-4778 (2022).

18. Burns, L. *et al.* Real-World Evidence for Regulatory Decision-Making: Guidance From Around the World. *Clin. Ther.* **44**, 420-437 (2022).

19. Canadian Agency for Drugs and Technologies in Health. *Use of real-world evidence in single-drug assessments*. <<https://www.cadth.ca/sites/default/files/pdf/es0323-rwe-in-single-drug-appraisal.pdf>> (2018). Accessed 24 February 2023.

20. Canadian Agency for Drugs and Technologies in Health. *Real-world evidence: A primer*. <<https://www.cadth.ca/real-world-evidence-primer>> (2023). Accessed 24 February 2023.

21. Cave, A., Kurz, X. & Arlett, P. Real-World Data for Regulatory Decision Making: Challenges and Possible Solutions for Europe. *Clin. Pharmacol. Ther.* **106**, 36-39 (2019).

22. Chan, K. *et al.* Developing a framework to incorporate real-world evidence in cancer drug funding decisions: the Canadian Real-world Evidence for Value of Cancer Drugs (CanREValue) collaboration. *BMJ Open* **10**, e032884 (2020).

23. Chen, J. *et al.* The Current Landscape in Biostatistics of Real-World Data and Evidence: Clinical Study Design and Analysis. *Stat. Biopharm. Res.* **15**, 29-42 (2023).

24. Crown, W., Dahabreh, I.J., Li, X., Toh, S. & Bierer, B. Can Observational Analyses of Routinely Collected Data Emulate Randomized Trials? Design and Feasibility of the Observational Patient Evidence for Regulatory Approval Science and Understanding Disease Project. *Value Health* **26**, 176-184 (2023).

25. de Lusignan, S., Crawford, L. & Munro, N. Creating and using real-world evidence to answer questions about clinical effectiveness. *J. Innov. Health. Inform.* **22**, 368-373 (2015).

26. Derman, B.A. *et al.* Reality check: Real-world evidence to support therapeutic development in hematologic malignancies. *Blood Rev.* **53**, 100913 (2022).

27. Dreyer, N.A. Advancing a Framework for Regulatory Use of Real-World Evidence: When Real Is Reliable. *Ther. Innov. Regul. Sci.* **52**, 362-368 (2018).

28. Dreyer, N.A., Hall, M. & Christian, J.B. Modernizing Regulatory Evidence with Trials and Real-World Studies. *Ther. Innov. Regul. Sci.* **54**, 1112-1115 (2020).

29. Duke-Margolis Center for Health Policy. *A framework for regulatory use of real-world evidence*. <<https://healthpolicy.duke.edu/sites/default/files/2020-08/rwe_white_paper_2017.09.06.pdf>> (2017). Accessed 24 February 2023.

30. Duke-Margolis Center for Health Policy. *Adding real-world evidence to a totality of evidence approach for evaluating marketed product effectiveness*. <<https://healthpolicy.duke.edu/sites/default/files/2020-08/Totality%20of%20Evidence%20Approach.pdf>> (2019). Accessed 24 February 2023.

31. Duke-Margolis Center for Health Policy. *Understanding the need for non-interventional studies using secondary data to generate real-world evidence for regulatory decision making, and demonstrating their credibility*. <<https://healthpolicy.duke.edu/sites/default/files/2020-08/Non-Interventional%20Study%20Credibility.pdf>> (2019). Accessed 24 February 2023.

32. Duke-Margolis Center for Health Policy. *Aligning shared evidentiary needs among payes and regulators for a real-world data ecosystem*. <<https://healthpolicy.duke.edu/sites/default/files/2022-07/RWE%20Aligning%20Shared%20Evidentiary%20Needs.pdf>> (2022). Accessed 24 February 2023.

33. Eichler, H.G. *et al.* Randomized Controlled Trials Versus Real World Evidence: Neither Magic Nor Myth. *Clin. Pharmacol. Ther.* **109**, 1212-1218 (2021).

34. Epstein, R.S., Sidorov, J., Lehner, J.P. & Salimi, T. Integrating scientific and real-world evidence within and beyond the drug development process. *J. Comp. Eff. Res.* **1**, 9-13 (2012).

35. Eskola, S.M., Leufkens, H.G.M., Bate, A., De Bruin, M.L. & Gardarsdottir, H. Use of Real-World Data and Evidence in Drug Development of Medicinal Products Centrally Authorized in Europe in 2018-2019. *Clin. Pharmacol. Ther.* **111**, 310-320 (2022).

36. European Medicines Agency. *Guidance for companies considering the adaptive pathways approach*. <<https://www.ema.europa.eu/en/documents/regulatory-procedural-guideline/guidance-companies-considering-adaptive-pathways-approach_en.pdf>> (2016). Accessed 24 February 2023.

37. European Medicines Agency. *Qualification opinion on The European Cystic Fibrosis Society Patient Registry (ECFSPR) and CF pharmaco-epidemiology studies*. <<https://www.ema.europa.eu/en/documents/regulatory-procedural-guideline/qualification-opinion-european-cystic-fibrosis-society-patient-registry-ecfspr-cf-pharmaco_en.pdf>> (2018). Accessed 24 February 2023.

38. European Medicines Agency. *Qualification opinion on Cellular therapy module of the European Society for Blood & Marrow Transplantation (EBMT) Registry*. <<https://www.ema.europa.eu/en/documents/scientific-guideline/qualification-opinion-cellular-therapy-module-european-society-blood-marrow-transplantation-ebmt_en.pdf>> (2019). Accessed 24 February 2023.

39. European Medicines Agency. *Guideline on registry-based studies*. <<https://www.ema.europa.eu/en/documents/scientific-guideline/guideline-registry-based-studies_en-0.pdf>> (2021). Accessed 24 February 2023.

40. European Network for Health Technology Assessment. *Position paper on how to best formulate research recommendations for primary research arising from HTA reports*. <<https://www.eunethta.eu/wp-content/uploads/2018/01/eunethta_position_paper_on_research_recommendations_0-1.pdf>> (2015). Accessed 24 February 2023.

41. Exley, A.R., Rantell, K. & McBlane, J. Clinical development of cell therapies for cancer: The regulators' perspective. *Eur. J. Cancer* **138**, 41-53 (2020).

42. Facey, K.M., Rannanheimo, P., Batchelor, L., Borchardt, M. & de Cock, J. Real-world evidence to support Payer/HTA decisions about highly innovative technologies in the EU-actions for stakeholders. *Int. J. Technol. Assess. Health Care*, 1-10 (2020).

43. Feinberg, B.A., Gajra, A., Zettler, M.E., Phillips, T.D., Phillips, E.G., Jr. & Kish, J.K. Use of Real-World Evidence to Support FDA Approval of Oncology Drugs. *Value Health* **23**, 1358-1365 (2020).

44. Flynn, R. *et al.* Marketing Authorization Applications Made to the European Medicines Agency in 2018-2019: What was the Contribution of Real-World Evidence? *Clin. Pharmacol. Ther.* **111**, 90-97 (2022).

45. Formica, M. The expanding role of real-world evidence in the regulatory environment. *Regulatory Rapporteur* **17**, 8-11 (2020).

46. Franklin, J.M., Glynn, R.J., Martin, D. & Schneeweiss, S. Evaluating the Use of Nonrandomized Real-World Data Analyses for Regulatory Decision Making. *Clin. Pharmacol. Ther.* **105**, 867-877 (2019).

47. Franklin, J.M., Liaw, K.L., Iyasu, S., Critchlow, C.W. & Dreyer, N.A. Real-world evidence to support regulatory decision making: New or expanded medical product indications. *Pharmacoepidemiol. Drug Saf.* **30**, 685-693 (2021).

48. Franklin, J.M. & Schneeweiss, S. When and How Can Real World Data Analyses Substitute for Randomized Controlled Trials? *Clin. Pharmacol. Ther.* **102**, 924-933 (2017).

49. Gajra, A., Zettler, M.E. & Feinberg, B.A. Randomization versus Real-World Evidence. *N. Engl. J. Med.* **383**, e21 (2020).

50. Garrison, L.P., Jr., Neumann, P.J., Erickson, P., Marshall, D. & Mullins, C.D. Using real-world data for coverage and payment decisions: the ISPOR Real-World Data Task Force report. *Value Health* **10**, 326-335 (2007).

51. George, E. How real-world data compensate for scarce evidence in HTA. *Z. Evid. Fortbild. Qual. Gesundhwes.* **112 Suppl 1**, S23-26 (2016).

52. Girman, C.J., Ritchey, M.E., McNeill, A.M., Sundell, K.A. & Meyer, R.J. Demonstrating that Real World Evidence Is Fit-For-Purpose to Support Labeling: Parallels to Patient Reported Outcomes in the Pursuit of Labeling Claims. *Ther. Innov. Regul. Sci.* **55**, 561-567 (2021).

53. Gross, A.M. Using real world data to support regulatory approval of drugs in rare diseases: A review of opportunities, limitations & a case example. *Curr. Probl. Cancer* **45**, 100769 (2021).

54. Hampson, G., Towse, A., Dreitlein, W.B., Henshall, C. & Pearson, S.D. Real-world evidence for coverage decisions: opportunities and challenges. *J. Comp. Eff. Res.* **7**, 1133-1143 (2018).

55. Health Canada. *Optimizing the use of real world evidence to inform regulatory decision-making*. <<https://www.canada.ca/en/health-canada/services/drugs-health-products/drug-products/announcements/optimizing-real-world-evidence-regulatory-decisions.html>> (2019). Accessed 24 February 2023.

56. Honig, P.K. The "Coming of Age" of Real-World Evidence in Drug Development and Regulation. *Clin. Pharmacol. Ther.* **111**, 11-14 (2022).

57. Institut National d'Excellence en Santé et en Services Sociaux. *Integration of real-world data and evidence to support decision-making in the pharmaceutical sector*. <<https://www.inesss.qc.ca/fileadmin/doc/INESSS/Rapports/Medicaments/INESSS_Real_world_data_SK.pdf>> (2022). Accessed 24 February 2023.

58. Institute for Clinical and Economic Review. *Real world evidence for coverage decisions: Opportunities and challenges. A report from the 2017 ICER membership policy summit*. <<https://icer.org/wp-content/uploads/2020/11/ICER-Real-World-Evidence-White-Paper-03282018.pdf>> (2018). Accessed 24 February 2023.

59. Institute for Clinical and Economic Review. *Considering clinical, real-world and unpublished evidence*. <<https://icer.org/our-approach/methods-process/considering-clinical-real-world-and-unpublished-evidence/>> (2023). Accessed 24 February 2023.

60. Institute of Health Economics. *Defining decision-grade real-world evidence and its role in the Canadian context: A design sprint*. <<https://www.ihe.ca/download/20181116_summary_report_realworldevidence_ihecaptcadthhealthcanada.pdf>> (2018). Accessed 24 February 2023.

61. Jaksa, A. *et al.* Key learnings from Institute for Clinical and Economic Review's real-world evidence reassessment pilot. *Int. J. Technol. Assess. Health Care* **38**, e32 (2022).

62. Jandhyala, R. A medicine adoption model for assessing the expected effects of additional real-world evidence (RWE) at product launch. *Curr. Med. Res. Opin.* **37**, 1645-1655 (2021).

63. Jarow, J.P., LaVange, L. & Woodcock, J. Multidimensional Evidence Generation and FDA Regulatory Decision Making: Defining and Using "Real-World" Data. *JAMA* **318**, 703-704 (2017).

64. Jonker, C.J., Bakker, E., Kurz, X. & Plueschke, K. Contribution of patient registries to regulatory decision making on rare diseases medicinal products in Europe. *Front. Pharmacol.* **13**, 924648 (2022).

65. Katkade, V.B., Sanders, K.N. & Zou, K.H. Real world data: an opportunity to supplement existing evidence for the use of long-established medicines in health care decision making. *J. Multidiscip. Healthc.* **11**, 295-304 (2018).

66. Kent, S. *et al.* The use of nonrandomized evidence to estimate treatment effects in health technology assessment. *J. Comp. Eff. Res.* **10**, 1035-1043 (2021).

67. Khosla, S. *et al.* Real world evidence (RWE) - a disruptive innovation or the quiet evolution of medical evidence generation? *F1000Res.* **7**, 111 (2018).

68. Kim, T.E., Park, S.I. & Shin, K.H. Incorporation of real-world data to a clinical trial: use of external controls. *Transl. Clin. Pharmacol.* **30**, 121-128 (2022).

69. Klonoff, D.C. The Expanding Role of Real-World Evidence Trials in Health Care Decision Making. *J. Diabetes Sci. Technol.* **14**, 174-179 (2020).

70. Klonoff, D.C. The New FDA Real-World Evidence Program to Support Development of Drugs and Biologics. *J. Diabetes Sci. Technol.* **14**, 345-349 (2020).

71. Klonoff, D.C., Gutierrez, A., Fleming, A. & Kerr, D. Real-World Evidence Should Be Used in Regulatory Decisions About New Pharmaceutical and Medical Device Products for Diabetes. *J. Diabetes Sci. Technol.* **13**, 995-1000 (2019).

72. Lamberti, M.J., Kubick, W., Awatin, J., McCormick, J., Carroll, J. & Getz, K. The Use of Real-World Evidence and Data in Clinical Research and Postapproval Safety Studies. *Ther. Innov. Regul. Sci.* **52**, 778-783 (2018).

73. Lau, C. & Dranitsaris, G. Impact of Regulatory Approval Status on CADTH Reimbursement of Oncology Drugs and Role of Real-World Evidence on Conditional Approvals from 2019 to 2021. *Curr. Oncol.* **29**, 8031-8042 (2022).

74. Lau, C., Jamali, F. & Loebenberg, R. Health Canada Usage of Real World Evidence (RWE) in Regulatory Decision Making compared with FDA/EMA usage based on publicly available information. *J. Pharm. Pharm. Sci.* **25**, 227-236 (2022).

75. Leahy, T.P., Ramagopalan, S. & Sammon, C. The use of UK primary care databases in health technology assessments carried out by the National Institute for health and care excellence (NICE). *BMC Health Serv. Res.* **20**, 675 (2020).

76. Levenson, M.S. Regulatory-grade clinical trial design using real-world data. *Clin. Trials* **17**, 377-382 (2020).

77. Liu, Q., Ramamoorthy, A. & Huang, S.M. Real-World Data and Clinical Pharmacology: A Regulatory Science Perspective. *Clin. Pharmacol. Ther.* **106**, 67-71 (2019).

78. Mahendraratnam, N., Mercon, K., Gill, M., Benzing, L. & McClellan, M.B. Understanding Use of Real-World Data and Real-World Evidence to Support Regulatory Decisions on Medical Product Effectiveness. *Clin. Pharmacol. Ther.* **111**, 150-154 (2022).

79. Maissenhaelter, B.E., Woolmore, A.L. & Schlag, P.M. Real-world evidence research based on big data: Motivation-challenges-success factors. *Onkologe (Berl)* **24**, 91-98 (2018).

80. Makady, A. *et al.* Policies for Use of Real-World Data in Health Technology Assessment (HTA): A Comparative Study of Six HTA Agencies. *Value Health* **20**, 520-532 (2017).

81. Makady, A. *et al.* Practical implications of using real-world evidence (RWE) in comparative effectiveness research: learnings from IMI-GetReal. *J. Comp. Eff. Res.* **6**, 485-490 (2017).

82. Makady, A. *et al.* Using Real-World Data in Health Technology Assessment (HTA) Practice: A Comparative Study of Five HTA Agencies. *Pharmacoeconomics* **36**, 359-368 (2018).

83. Malone, D.C. Real-world evidence enhances decision making. *J. Manag. Care Spec. Pharm.* **26**, 1612-1614 (2020).

84. Miksad, R.A. & Abernethy, A.P. Harnessing the Power of Real-World Evidence (RWE): A Checklist to Ensure Regulatory-Grade Data Quality. *Clin. Pharmacol. Ther.* **103**, 202-205 (2018).

85. Mofid, S., Bolislis, W.R. & Kuhler, T.C. Real-World Data in the Postapproval Setting as Applied by the EMA and the US FDA. *Clin. Ther.* **44**, 306-322 (2022).

86. Mospan, A.R., Morris, H.L. & Fried, M.W. Real-world evidence in hepatocellular carcinoma. *Liver Int.* **41 Suppl 1**, 61-67 (2021).

87. National Institute for Health and Care Excellence. *NICE real-world evidence framework*. <<https://www.nice.org.uk/corporate/ecd9/resources/nice-realworld-evidence-framework-pdf-1124020816837>> (2022). Accessed 24 February 2023.

88. Naumann-Winter, F. *et al.* Licensing of Orphan Medicinal Products-Use of Real-World Data and Other External Data on Efficacy Aspects in Marketing Authorization Applications Concluded at the European Medicines Agency Between 2019 and 2021. *Front. Pharmacol.* **13**, 920336 (2022).

89. Nazha, B., Yang, J.C. & Owonikoko, T.K. Benefits and limitations of real-world evidence: lessons from EGFR mutation-positive non-small-cell lung cancer. *Future Oncol.* **17**, 965-977 (2021).

90. Nicotera, G., Sferrazza, G., Serafino, A. & Pierimarchi, P. The Iterative Development of Medicines Through the European Medicine Agency's Adaptive Pathway Approach. *Front. Med. (Lausanne)* **6**, 148 (2019).

91. O'Donnell, J.C. *et al.* Evolving use of real-world evidence in the regulatory process: a focus on immuno-oncology treatment and outcomes. *Future Oncol.* **17**, 333-347 (2021).

92. Olson, M.S. Can real-world evidence save pharma US$1 billion per year? A framework for an integrated evidence generation strategy. *J. Comp. Eff. Res.* **9**, 79-82 (2020).

93. Polak, T.B., van Rosmalen, J. & Uyl-de Groot, C.A. Expanded Access as a source of real-world data: An overview of FDA and EMA approvals. *Br. J. Clin. Pharmacol.* **86**, 1819-1826 (2020).

94. Pulini, A.A., Caetano, G.M., Clautiaux, H., Vergeron, L., Pitts, P.J. & Katz, G. Impact of Real-World Data on Market Authorization, Reimbursement Decision & Price Negotiation. *Ther. Innov. Regul. Sci.* **55**, 228-238 (2021).

95. Purpura, C.A., Garry, E.M., Honig, N., Case, A. & Rassen, J.A. The Role of Real-World Evidence in FDA-Approved New Drug and Biologics License Applications. *Clin. Pharmacol. Ther.* **111**, 135-144 (2022).

96. Ramamoorthy, A. & Huang, S.M. What Does It Take to Transform Real-World Data Into Real-World Evidence? *Clin. Pharmacol. Ther.* **106**, 10-18 (2019).

97. Raphael, M.J., Gyawali, B. & Booth, C.M. Real-world evidence and regulatory drug approval. *Nat. Rev. Clin. Oncol.* **17**, 271-272 (2020).

98. Schad, F. & Thronicke, A. Real-World Evidence-Current Developments and Perspectives. *Int. J. Environ. Res. Public Health* **19**, (2022).

99. Schneeweiss, S. & Glynn, R.J. Real-World Data Analytics Fit for Regulatory Decision-Making. *Am. J. Law Med.* **44**, 197-217 (2018).

100. Sherman, R.E. *et al.* Real-World Evidence - What Is It and What Can It Tell Us? *N. Engl. J. Med.* **375**, 2293-2297 (2016).

101. Sievers, H., Joos, A. & Hiligsmann, M. Real-world evidence: perspectives on challenges, value, and alignment of regulatory and national health technology assessment data collection requirements. *Int. J. Technol. Assess. Health Care* **37**, e40 (2021).

102. Song, F. *et al.* The use of real-world data/evidence in regulatory submissions. *Contemp. Clin. Trials* **109**, 106521 (2021).

103. Spitzer, E., Cannon, C.P. & Serruys, P.W. Should real-world evidence be incorporated into regulatory approvals? *Expert Opin. Drug Saf.* **17**, 1155-1159 (2018).

104. Tadrous, M., Ahuja, T., Ghosh, B. & Kropp, R. Developing a Canadian Real-World Evidence Action Plan across the Drug Life Cycle. *Healthc. Policy* **15**, 41-47 (2020).

105. US Food and Drug Administration. *Framework for FDA's Real-World Evidence Program*. <<https://www.fda.gov/media/120060/download>> (2018). Accessed 24 February 2023.

106. US Food and Drug Administration. *Use of electronic health record data in clinical investigations. Guidance for industry*. <<https://www.fda.gov/media/97567/download>> (2018). Accessed 24 February 2023.

107. US Food and Drug Administration. *Real-world data: Assessing registries to support regulatory decision-making for drug and biological products. Guidance for industry*. <<https://www.fda.gov/media/154449/download>> (2021). Accessed 24 February 2023.

108. US Food and Drug Administration. *Submitting documents using real-world data and real-world evidence to FDA for drug and biological products. Guidance for industry*. <<https://www.fda.gov/media/124795/download>> (2022). Accessed 24 February 2023.

109. US Food and Drug Administration. *Considerations for the design and conduct of externally controlled trials for drug and biological products. Guidance for industry*. <<https://www.fda.gov/media/164960/download>> (2023). Accessed 24 February 2023.

110. Varnai, P., Dave, A., Farla, K., Nooijen, A. & Petrosova, L. The Evidence REVEAL Study: Exploring the Use of Real-World Evidence and Complex Clinical Trial Design by the European Pharmaceutical Industry. *Clin. Pharmacol. Ther.* **110**, 1180-1189 (2021).

111. Wallach, J.D., Ross, J.S. & Naci, H. The US Food and Drug Administration's expedited approval programs: Evidentiary standards, regulatory trade-offs, and potential improvements. *Clin. Trials* **15**, 219-229 (2018).

112. Webster, J. & Smith, B.D. The Case for Real-world Evidence in the Future of Clinical Research on Chronic Myeloid Leukemia. *Clin. Ther.* **41**, 336-349 (2019).

113. Wu, J., Wang, C., Toh, S., Pisa, F.E. & Bauer, L. Use of real-world evidence in regulatory decisions for rare diseases in the United States-Current status and future directions. *Pharmacoepidemiol. Drug Saf.* **29**, 1213-1218 (2020).

114. Yap, T.A., Jacobs, I., Baumfeld Andre, E., Lee, L.J., Beaupre, D. & Azoulay, L. Application of Real-World Data to External Control Groups in Oncology Clinical Trial Drug Development. *Front. Oncol.* **11**, 695936 (2021).

115. Zettler, M.E. The use of real-world evidence to support FDA post-approval study requirements for oncology drugs. *Expert Rev. Anticancer Ther.* **22**, 657-666 (2022).

116. Zorginstituut Nederland. *Richtlijn voor het uitvoeren van economische evaluaties in de gezondheidszorg*. <<https://www.zorginstituutnederland.nl/binaries/zinl/documenten/publicatie/2016/02/29/richtlijn-voor-het-uitvoeren-van-economische-evaluaties-in-de-gezondheidszorg/richtlijn-voor-het-uitvoeren-van-economische-evaluaties-in-de-gezondheidszorg.pdf>> (2016). Accessed 24 February 2023.

117. Zou, K.H. *et al.* Harnessing Real-World Data for Regulatory Use and Applying Innovative Applications. *J. Multidiscip. Healthc.* **13**, 671-679 (2020).

118. Zura, R., Irwin, D.E., Mack, C.D., Aldridge, M.L. & Mackowiak, J.I. Real-World Evidence: A Primer. *J. Orthop. Trauma* **35**, S1-S5 (2021).
